# Underlying MASLD-induced gut microbiome dysbiosis and intestinal inflammation are key to poor outcomes in vibriosis infections in a preclinical model

**DOI:** 10.1080/19490976.2026.2652474

**Published:** 2026-04-13

**Authors:** Punnag Saha, Subhajit Roy, Madhura More, Dipro Bose, Ayushi Trivedi, Bryan W. Brooks, Wing-Kin Syn, Anna Mae Diehl, Saurabh Chatterjee

**Affiliations:** a Department of Environmental and Occupational Health, Environmental Health and Disease Laboratory, Joe C. Wen School of Population and Public Health, University of California, Irvine, CA, USA; b Department of Pediatrics, University of California San Diego, La Jolla, CA, USA; c Department of Environmental Science, Baylor University, Waco, TX, USA; d Department of Internal Medicine, Division of Gastroenterology and Hepatology, Saint Louis University School of Medicine, St. Louis, MO, USA; e Department of Physiology, Faculty of Medicine and Nursing, University of Basque Country UPV/EHU, Vizcaya, Spain; f Department of Pathology, Saint Louis University, St Louis, MO, USA; g Division of Gastroenterology, Duke University School of Medicine, Durham, NC, USA; h Department of Environmental and Occupational Health, Toxicology Core, NIEHS Center for Oceans and Human Health on Climate Change Interactions, Program in Public Health, University of California, Irvine, CA, USA; i Department of Medicine, Division of Infectious Disease, UCI School of Medicine, University of California, Irvine, CA, USA

**Keywords:** MASLD, *Vibrio vulnificus*, non-cholera vibriosis, gut microbiome, inflammation, fecal microbiota transplantation

## Abstract

Metabolic dysfunction-associated steatotic liver disease (MASLD) is the leading cause of chronic liver disease globally, especially in developed countries, including the United States. The etiology of MASLD is closely associated with several other cardiometabolic conditions and can further aggravate to more severe stages of liver disease, including steatohepatitis and cirrhosis. Moreover, patients with underlying MASLD conditions have altered gut microbiome signatures and intestinal homeostasis, leading to gut barrier dysfunction, thereby making them more vulnerable to acute gastrointestinal infections like non-cholera vibriosis. However, the exact role of the gut microbiome and intestinal pathophysiology in increasing susceptibility to infection in patients with MASLD remains poorly understood. In this study, we used oral inoculation of the bacterium *Vibrio vulnificus* to investigate the pathophysiological outcomes in both control and diet-induced MASLD mouse cohorts. Our results showed that non-cholera vibriosis in mice with underlying MASLD caused increased liver damage, an inflammatory surge, followed by the onset of fibrotic lesions compared to the chow-diet fed control mice, depicting a worsened outcome. Depletion of the gut bacteriome by antibiotic treatment and following fecal microbiota transplantation in these mouse cohorts showed decreased pathophysiology in the livers, indicating that an altered gut microbiome in MASLD could be a key factor in the increased likelihood of non-cholera vibriosis in patients with MASLD.

## 1. Introduction

MASLD [previously known as nonalcoholic fatty liver disease (NAFLD)] is the most prevalent form of underlying chronic liver disease worldwide, with an annual incidence of 46.9 cases per 1000 persons.^1^ The disease spectrum of MASLD ranges from a benign steatosis stage to advanced and fatal forms like cirrhosis and hepatocellular carcinoma.^1^ Furthermore, patients with MASLD often have several other cardiometabolic conditions such as obesity, insulin resistance, type 2 diabetes mellitus, dyslipidemia, and hypertension.^2^ Apart from these pathological conditions, several recent studies have indicated that patients with underlying chronic liver disease conditions are at increased risk of serious nosocomial infections as well as recurrent bacterial infections.^3^ In particular, a recently published study by Patel et al. also reported that patients with MASLD are more likely to be susceptible to various gastrointestinal infections, as a higher prevalence of these gastrointestinal bacterial infections (by *Clostridioides difficile*, *Escherichia coli*, and *Salmonella*) was found in these patients compared to patients without MASLD.^4^ Apart from causing pathological changes in the liver, MASLD also results in significant alterations in intestinal homeostasis and the gut microbiome in individuals via the gut–liver axis, which could be associated with the increased risk of these gastrointestinal infections.^5^

Non-cholera vibriosis caused by the bacteria *Vibrio vulnficus* (VV*)* and *Vibrio parahaemolyticus* is one of the major foodborne infection diseases reported worldwide, with a global estimate of around half a million cases in 2020.^6^ In the United States alone, data from both the Cholera and Other Vibrio Illness Surveillance (COVIS) system and the 10-state Foodborne Diseases Active Surveillance Network (FoodNet) reported that annual incidences regarding non-cholera vibriosis per 100,000 population increased drastically from 1996 to 2010 (0.09–0.28 in COVIS and 0.15–0.42 in FoodNet).^7^ Many reports have indicated that climate change-mediated global warming, warmer seawater temperatures, and low salinity are some of the key contributing factors regarding the increased occurrences of non-cholera vibriosis cases worldwide.^8^^,^^9^ The persistence of these conditions in the marine aquatic systems greatly favors the growth of these non-cholera *Vibrio* species, resulting in their increased abundance in both seawater and seafood. Among the other non-cholera *Vibrio* species, VV is a notorious human pathogen known for its highest food-borne fatality rate.^10^ Humans can be exposed to VV via dermal or orogastric routes. Dermal exposure to VV occurs mainly during recreational activities (e.g., swimming, fishing, and handling seafood), and can then lead to severe wound infection and possibly necrotizing fasciitis, even resulting in amputation.^11^ In parallel, oral exposure to VV mainly occurs via the consumption of contaminated raw or undercooked molluscan shellfish (primarily oysters) and may cause severe gastroenteritis and septicemia, leading to death.^12^

Many studies and clinical cases have previously indicated that pre-existing chronic liver disease conditions can serve as a major risk factor for vibriosis severity in patients.^13-15^ Very recently, a study predicted that patients with chronic liver diseases are at a 3-fold increased risk of VV infection, whereas patients with MASLD and cirrhosis are at a 2-fold and 5-fold increase, respectively, according to univariate regression.^16^ However, the role of the host’s gut microbiome, along with the possible underlying factors in MASLD pathophysiology that could contribute to this increased susceptibility to non-cholera vibriosis, remains unexplored. Therefore, we aimed to establish the role of the gut microbiome and identify the key contributing factors that can lead to increased vibriosis-related pathophysiological outcomes in the present study using a murine model of MASLD.

## 2. Materials and methods

### 2.1. Materials

Ampicillin, metronidazole, neomycin, vancomycin, and all other chemicals were purchased from Sigma–Aldrich (St. Louis, MO, USA) unless otherwise specified. All antibodies used in this study are listed in Supplementary Table 1.

### 2.2. Mice

Pathogen-free, wild-type, adult (14 weeks old), male C57BL/6J mice were acquired from Jackson Laboratories (Bar Harbor, ME, USA). Upon arrival, all the mice were housed inside the vivarium (22–24 °C with a 12 h light/12 h dark cycle) and had ad libitum access to food and water. A randomization procedure was performed to allocate the mice to the cages (3 mice/cage). All animal experiments for this study were approved by the University of California Irvine Institutional Animal Care and Use Committee (IACUC), complied with the NIH guidelines for the humane care and use of laboratory animals, and the Animal Research: Reporting of In Vivo Experiments (ARRIVE) guidelines.

To limit microbiome differences arising from cage effects, the mice were first ranked according to body weight and then distributed across three cages per treatment arm using a stratified randomization strategy, as previously outlined in the existing literature.^17^ This approach was applied consistently in both conventional and post-fecal microbiota transplantation (FMT) settings. The animals were rotated between cages within the same group twice a week, and tail markings were used to maintain reliable identification over time.

All mice were humanely euthanized via CO_2_ inhalation (30%–70% chamber volume displacement rate). Following euthanasia, blood was collected by the cardiac puncture method. Fresh serum samples were obtained and stored immediately at −80 °C. The livers and small intestines were also harvested, fixed in 10% neutral buffered formalin, and sent to the University of California Irvine School of Medicine's Experimental Tissue Resource for paraffin embedding and sectioning. Fecal pellets were collected, immediately flash-frozen in liquid nitrogen, and stored at −80 °C until shipment to CosmosID Inc. (Germantown, MD, USA) for microbiome and resistome analysis within one week. Strict measures were implemented to avoid any freeze‒thaw cycles of samples during this process.

### 2.3. Experimental murine model of diet-induced MASLD

Upon arrival at the vivarium, all mice were allowed to acclimate for 1 week. All mice (aged 15 weeks) were randomly divided into two cohorts (LEAN and MASLD). All mice from the LEAN cohort were fed a chow diet, whereas mice from the MASLD cohort were fed a choline-deficient high-fat diet (CD-HFD) (L-amino acid diet with 60 kcal% fat, 0.1% methionine, and no added choline, irradiated) [Catalog No. A06071302i; Research Diets, New Brunswick, NJ, USA] for 20 weeks until the completion of the study.

### 2.4. *Vibrio vulnificus* culture and oral administration

*Vibrio vulnificus* strain 324 [strain designation: CDC B9629] was purchased from ATCC [Catalog No. 27562™] and sub-cultured in Marine Agar 2216 or marine broth 2216 (BD DIFCO™, Franklin Lakes, NJ, USA) at 30 °C following the vendor’s recommendations. An overnight-grown VV starter culture was diluted (1:10) in pre-warmed marine broth, incubated at 30 °C, and shaken at 200  rpm until the optical density at 600 nm (OD_600_) reached 0.5–0.6, indicating mid-log phase growth of the bacteria. The bacterial cells were subsequently harvested by centrifugation at 13,800 × g for 10 min at room temperature. The bacterial pellet was resuspended in sterile phosphate-buffered saline (PBS) containing 0.01% (w/v) gelatin and further diluted to prepare a stock solution of 10^9^ CFU/mL.^18^

For our intragastric infection model, the mice were fasted without food and water for at least 4 h before VV administration. Before the oral inoculation, mice were first administered 50  μL of 10% (w/v) sodium bicarbonate. After 15 min, mouse groups from the LEAN cohort (LEAN + VV) or the MASLD cohort (MASLD + VV) were orally administered the VV inoculum (10^8^ CFU/100 µL) in PBS containing 0.01% (w/v) gelatin. Food and water were provided back to the mice 30 min post-inoculation, and the mice were euthanized after 24  h.

### 2.5. Depletion of the gut microbiome by antibiotic cocktail and fecal microbiota transplantation

We used an antibiotic cocktail (ABX) consisting of ampicillin, metronidazole, neomycin (1 g/kg body weight; dissolved in PBS), and vancomycin (0.5 g/kg body weight; dissolved in PBS) to deplete the murine gut microbiome of the experimental mice as described in a prior study from our research group^19^ with further modifications.^20^ Then, 100 µL of the ABX cocktail was orally administered to LEAN mice (LEAN + ABX) and MASLD mice (MASLD + ABX) cohorts consecutively for 15 d.

For fecal microbiota transplantation (FMT), fresh fecal solution samples were prepared daily before administration of the dose. Fresh fecal samples (approximately 150 mg) were collected daily from healthy donor mice from the LEAN Control. The feces were subsequently diluted in 1 mL of sterile PBS and centrifuged at 3000 × g for 5 min, after which the supernatants were collected. Then, 100  µL of the supernatant was orally administered to the ABX-pretreated LEAN cohort (LEAN + FMT + VV) and MASLD cohort (MASLD + FMT + VV) consecutively for 7 d. Upon completion of FMT treatment, all the mice were intragastrically administered with the VV inoculum for 24 h.

### 2.6. Analysis of gut bacteriome

Raw reads and taxonomic results were generated by the vendor CosmosID, Inc., as mentioned in other studies.^21^^,^^22^ Using the fecal pellets from both the LEAN and MASLD mouse groups, DNA was extracted via the QIAGEN DNeasy PowerSoil Pro Kit following the manufacturer's instructions. The concentration of the purified DNA samples was then measured using the Qubit 4 fluorometer with the Qubit™ dsDNA HS Assay Kit (Thermo Fisher Scientific, Waltham, MA, USA). Then, 1 ng of the extracted DNA served as input for library preparation, which was completed using the Nextera XT DNA Library Preparation Kit (Illumina) along with IDT Unique Dual Indexes. Library construction was performed by fragmenting gDNA with the Illumina Nextera XT fragmentation enzyme. Unique dual indices were then ligated, followed by a 12-cycle PCR amplification. The resulting DNA libraries were subsequently purified using AMpure magnetic beads (Beckman Coulter) and eluted in QIAGEN EB buffer. After that, the Qubit 4 fluorometer and a Qubit™ dsDNA HS Assay Kit were used to quantify the DNA libraries. Sequencing of the libraries was conducted on an Illumina NovaSeq 6000 platform at 2x150 bp.

The system employs a fast, two-step, k-mer-based data-mining pipeline that first creates unique genomic fingerprints from curated databases and then screens millions of sample sequences against those fingerprints for sensitive, high-precision taxonomic classification and the detection of virulence and resistance genes, all while using strict statistical filters to ensure accuracy. Analysis of the statistics provided detailed taxonomic and relative abundance estimates for the microbial NGS datasets. To eliminate false positive identification, the results were filtered using a statistically derived threshold based on scores from diverse metagenome analyses.

### 2.7. Identification and analysis of the gut resistome

Bacterial antibiotic resistance genes (ARGs) were also identified in the fecal pellets from the LEAN and MASLD mouse groups, using the CosmosID platform as mentioned in our earlier study.^23^ Raw metagenomic sequencing reads were first quality-filtered with fastp (fastp_qualified_quality of 15 and fastp_cut_mean_quality of 15). The trimmed reads were then assembled using MEGAHIT with default parameters to generate metagenome-assembled genomes (MAGs) through MetaBAT2 binning with default parameters. The quality assessment of the MAGs was confirmed using QUAST and BUSCO with default parameters. The MAGs were then processed using the CosmosID-Hub Microbiome Platform for taxonomic identification and the CosmosID ARG pipeline for ARG identification. The pipeline screened the assembled genomes against the ResFinder ARG database for ARGs. ARGs were determined to be present using a strict cutoff (>90 nucleotide identity and > 60% alignment coverage against the gene's sequence length). All MAGs were subsequently processed through the CosmosID-HUB Microbiome Platform for comprehensive profiling of ARGs as described in earlier findings.^24^

### 2.8. Histopathology and staining

Formalin-fixed, paraffin-embedded liver tissue slices (5 μm) were deparaffinized using a standard, established protocol as mentioned in our earlier studies.^25^^,^^26^ Briefly, liver slices were immersed in solutions of 100% xylene, a 1:1 solution of xylene and ethanol, 100% ethanol, 95% ethanol, 70% ethanol, 50% ethanol, and deionized water in a sequential manner. The liver sections were then stained with hematoxylin and eosin (H&E) and picrosirius red (PSR) (to detect collagen deposition) following the manufacturer's directions (Catalog No. IW-3012, NovaUltra Special Stain Kits, IHC World, Woodstock, MD). Images of the stained sections were captured using an Olympus BX43 microscope at 100× and 200× magnification.

For Alcian blue periodic acid-Schiff (PAS) staining, we used a commercially available kit (Catalog No. AB245876, Abcam, Cambridge, MA, USA). Small intestine tissue sections were deparaffinized first, as mentioned above. After 3% acetic acid treatment, the tissues were stained with Alcian Blue (pH 2.5) for 15‒20 min. Then, 1% periodic acid solution was added for 5 min, followed by the addition of Schiff’s solution for 10–20 min. Modified Mayer's hematoxylin solution was applied to the sections for 2 min. After the final rinse, all the tissue sections were dehydrated using a series of graded alcohol solutions. Finally, mounting was completed using synthetic resin to preserve the sample for microscopic examination.

### 2.9. NAFLD activity score and fibrosis score assessment

Liver histology was examined in all the mouse groups using H&E-stained sections, and the NAFLD activity score (NAS) was assessed in a blinded manner. The NAS (range 0–8) was calculated by adding the scores for steatosis (0–3), lobular inflammation (0–3), and hepatocyte ballooning (0–2).^27^ Additionally, the fibrosis score (stage 0–4) was determined from PSR-stained liver sections.^27^

### 2.10. Serum alanine aminotransferase (ALT), aspartate aminotransferase (AST) measurements

Serum levels of ALT (Catalog No. 700260, Cayman Chemical, Ann Arbor, MI) and AST (Catalog No. 701640, Cayman Chemical, Ann Arbor, MI) were measured following the manufacturer’s instructions.

### 2.11. Immunohistochemistry

Formalin-fixed, paraffin-embedded tissue slices (5 μm) from the liver or small intestine were first deparaffinized by our standard laboratory procedure. Then, the antigen retrieval was conducted using the Epitope retrieval solution and a steamer (Catalog No. IW-1100, IHC-World, Woodstock, MD, USA). Next, endogenous peroxidase blocking (using 3% H_2_O_2_ solution) and serum blocking (with 5% goat serum) were performed. After that, anti-*α*-SMA, anti-IL-1β, anti-TGF-β1, and anti-fibronectin primary antibodies were applied (1:300 dilution) to the sections, which were subsequently incubated overnight at 4 °C in a humidified chamber. All tissue sections were washed 3 times using PBS supplemented with 0.05% Tween 20. Then, the sections were probed with species-specific biotinylated secondary antibodies (1:250 dilution), followed by incubation with a Vectastain Elite ABC-HRP Kit. (Catalog No. PK-6100, Vector Laboratories, Inc., Newark, CA). 3,3-diaminobenzidine chromogenic substrate solution (Catalog No. AB64238, Abcam, Cambridge, MA, USA) was used to stain the sections, whereas Mayer’s hematoxylin was utilized as a counterstain. Finally, the sections were mounted with Simpo mount (GBI Laboratories, Mukilteo, WA, USA). The immunoreactivity in the sections was captured by an Olympus BX63 microscope at 200× magnification.

### 2.12. Immunofluorescence staining

Formalin-fixed, paraffin-embedded small intestine tissue sections were also subjected to deparaffinization and epitope retrieval procedures as described previously. Next, the sections were permeabilized with 0.1% Triton X-100 in PBS and blocked with 5% goat serum. Primary antibodies targeting ZO-1, Occludin, Claudin-2, and MUC2 were applied (1:300) and incubated overnight at 4 °C in a humidified chamber. Subsequent incubation with Alexa Fluor 488/633 secondary antibodies (1:250 dilution) was performed. Finally, the sections were mounted with ProLong™ Diamond Antifade Mountant with DAPI (Catalog No. P36971, Invitrogen, Waltham, MA, USA). All images were acquired at 400 ×  magnification with an Olympus BX63 microscope.

### 2.13. Morphometric analyses by CellSens software

All morphometric analyses of the stained images (immunohistochemistry and immunofluorescence) were completed in a blinded fashion using the Count and Measure feature of Olympus CellSens Software from Olympus America (Center Valley, PA, USA). Identical imaging conditions were maintained across all samples for parameters such as magnification, exposure time, and gain to ensure consistency. The process involved selecting the target areas as regions of interest (ROI), which were either DAB-positive signals or colocalized fluorescent dots. Then, adaptive thresholding was used to semi-automatically quantify the percentage of the selected ROI regions relative to the total image area.

### 2.14. Isolation of liver nonparenchymal cells

Primary hepatic macrophages were isolated from the experimental mice following the protocol described by Daemen et al.^28^ Post-euthanization, cardiac perfusion was performed to remove excess blood, and approximately one-third of the liver sections were finely minced and collected in tubes containing digestion buffer (DMEM supplemented with collagenase A and DNase I). After incubation for 30 min at 37 °C, cold DMEM complete media (supplemented with 10% FBS, 100 mM sodium pyruvate, and 100 mM L-glutamine) was added to stop the enzymatic activity, and the digested liver mixtures were then passed through 70 μm cell strainers. After that, the strained cell suspensions were first centrifuged at 50 × g for 3 min at 4 °C, and the supernatants containing non-parenchymal cells (NPCs) were collected. Next, the cell suspensions were further centrifuged at 163 × g for 7  min at 4 °C to pellet down the NPCs, and 1x red blood cell (RBC) lysis buffer (Catalog No. 420301, BioLegend, San Diego, CA, USA) was added to remove any traces of erythrocytes. Finally, the isolated NPCs were resuspended in cold PBS and collected in tubes.

### 2.15. Flow cytometry

Isolated liver NPCs were washed with BD Pharmingen™ Stain Buffer (Catalog No. 565853, BD Biosciences, Franklin Lakes, New Jersey, USA) and resuspended at a concentration of 1 × 10^6^ cells/100 μL. Then, the cells were stained for viability by incubation with live/dead fixable near-IR viability dye (Catalog No. 420403, BioLegend, San Diego, CA, USA) for 20 min on ice in the dark. Following two washes, fixation was performed with a 1x True-Nuclear™ Transcription Factor Buffer Set (Catalog No. 424401, BioLegend, San Diego, CA, USA) for 15 min at room temperature in the dark. Then, the cells were blocked with TruStain FcX™ antibody (Catalog No. 101319, BioLegend, San Diego, CA, USA) for 10 min at 4 °C. Consequently, the cells were stained with specific antibodies, including BUV395 rat anti-mouse CD45, V450 rat anti-CD11b, BV711 rat anti-mouse F4/80, and PE anti-mouse CD86 at 4 °C for 20 min. The cells were then washed three times with the BD Pharmingen™ Stain Buffer, and flow cytometry was performed using a BD Fortessa™ flow cytometer (BD Biosciences, Franklin Lakes, New Jersey, USA). Finally, the analysis was performed using FlowJo software v10 from BD Biosciences (Ashland, OR, USA).

### 2.16. Enzyme-linked immunosorbent assay (ELISA)

Serum hepcidin (ng/mL) (Catalog No. NBP2-82129, Novus Biologicals, Centennial, CO, USA), ferritin (μg/mL) (Catalog No. KA1941, Novus Biologicals, Centennial, CO, USA), immunoglobulin-A (IgA) (μg/mL) (Catalog No. ab157717, Abcam, Cambridge, MA, USA) and C-reactive protein (CRP) (μg/mL) (Catalog No. MCRP00, Novus Biological, Centennial, CO, USA) were quantified as per the manufacturers’ instructions using commercially available ELISA kits. For the measurement of butyrate in the feces, we utilized a commercially available kit (Catalog No. abx258338, Abbexa, Cambridge, United Kingdom). Fecal pellets were first homogenized using an extraction buffer (1x PBS + 0.1% Tween-20). After centrifugation (12,000xg at 4 °C for 5 min), the supernatants were collected and freshly used for the assay.

### 2.17. Endotoxemia detection by limulus amebocyte lysate (LAL) assay

The endotoxin concentration (EU/mL) in the serum was measured using the Pierce LAL Chromogenic Endotoxin Quantitation Kit (Catalog No. A39552, Thermo Fisher Scientific, Waltham, MA, USA), following the manufacturer's protocol.

### 2.18. Quantitative real-time polymerase chain reaction

DNA extraction from the fecal pellets was performed via the same method as mentioned earlier. Using SsoAdvanced SYBR Green Supermix (Bio-Rad, Hercules, CA, USA), quantitative real-time PCR (qRT-PCR) was performed on a CFX96 thermal cycler (Bio-Rad, Hercules, CA, USA). For the detection of *V. vulnificus*, primers targeting *vvhA* (hemolysin A) were utilized: forward 5′-TTCCAACTTCAAACCGAACTATGA-3′ and reverse 5′-ATTCCAGTCGATGCGAATACGTTG-3′. Additionally, the bacterial load in the feces was also quantified using Universal 16S rRNA gene primers: forward 5′-GGGCTACACACGYGCWAC-3′ and reverse 5′-GACGGGCGGTGTGTRCA-3′ as previously validated by our group.^29^ The relative abundance was calculated using the ΔCt method by normalizing to universal 16S rRNA levels. For analysis, average Ct values were determined from technical triplicates of all reactions.

### 2.19. Network analysis

To visualize host–microbiome–immune interactions associated with MASLD, we constructed a directed functional network using the R igraph package as mentioned in our earlier study.^29^ Nodes in the network represented bacterial taxa, immune or barrier markers, intermediate mechanisms (e.g., SCFA or mucin production), and disease outcomes (e.g., fibrosis, inflammation, and MASLD progression). The bacterial nodes were categorized as MASLD-associated, beneficial, or neutral based on relative abundance data and literature-supported functional roles. Immune markers are present in both the small intestinal and hepatic compartments (e.g., IL-1β, ZO-1, CD86, and *α*-SMA), while functional nodes capture barrier integrity and immune polarization. Directed edges represent known or inferred biological influences – e.g., microbial induction of SCFA production or immune cytokines driving inflammation – and are weighted (0.6–0.95) to reflect the relative strength of evidence. Nodes were styled by type (shape and size) and colored according to functional cluster, and a Fruchterman–Reingold force-directed layout with high node repulsion was used to optimize spatial separation. Custom label offsets and legends were added to enhance interpretability, and the final network was plotted to depict microbial contributions and immune mediators driving MASLD-associated mucosal and hepatic dysfunction.

### 2.20. Statistical analyses

All statistical analyses for this study were performed using GraphPad Prism software version 10.4.0 (San Diego, CA, USA), and the data are presented as mean ± SEM. For determining inter-group comparison, Unpaired t-test (two-tailed tests with equal variance) and one-way analysis of variance (ANOVA) were performed, followed by Bonferroni post-hoc corrections analysis. *α*-Diversity was analyzed using several metrics to compare the microbial richness and evenness within different samples. These calculations were performed in R using the Vegan package. To determine if there were significant differences in diversity between the groups, Wilcoxon rank sum tests were used and represented by box and whisker plots, which were performed with the ggsignif package. For *β*-diversity, the Bray‒Curtis dissimilarity was calculated using the vegdist function of the R package vegan. To visualize these differences, principal coordinate analysis (PCoA) plots were generated with the PCoA function from the ape package. To statistically test for significant differences in community composition, PERMANOVA was performed using the adonis function from vegan. Finally, all plots were created using the ggpubr package in R. For all analyses, *p* ≤ 0.05 (*p < 0.05, **p < 0.01, ***p < 0.001) was considered statistically significant.

## 3. Results

### 3.1. Feeding with CD-HFD for 20 weeks imparts MASLD pathophysiology in mice

**Figure 1.**
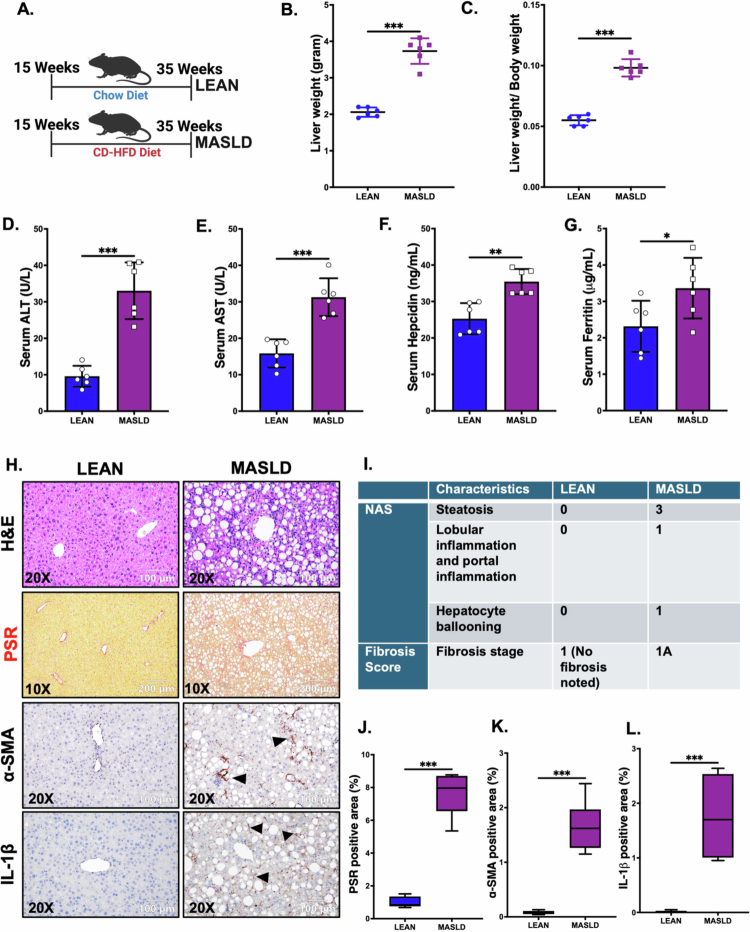
Establishment of MASLD pathophysiology in mice by CD-HFD feeding for 20 weeks. (A) Schematic representation of the experimental mouse groups: LEAN [*n* = 6; chow diet-fed mice for 20 weeks] and MASLD [*n* = 6; CD-HFD fed mice for 20 weeks]. (B) Liver weight (grams), and (C) liver-to-body weight ratio of the LEAN and MASLD mouse groups. Serum levels of (D) ALT (U/L), (E) AST (U/L), (F) hepcidin (ng/mL), and (G) ferritin (μg/mL) in the LEAN and MASLD groups. Formalin-fixed, paraffin-embedded 5 μm liver slices from the LEAN and MASLD groups were used for histopathological analyses. Representative images of (H) hematoxylin and eosin (H&E) staining, picrosirius red (PSR) staining, and immunohistochemistry images depicting *α*-SMA, and IL-1β immunoreactivity (indicated by black arrowheads) in the liver sections of LEAN and MASLD mouse groups. H&E and immunohistochemistry images were captured at 200× magnification, whereas PSR images were captured at 100× magnification. (I) NAFLD activity score (NAS) and fibrosis score for the LEAN and MASLD groups. Morphometric analyses (calculated as %ROI) of (J) PSR staining, (K) *α*-SMA, and (L) IL-1β immunoreactivity, where the Y-axis represents % positive immunoreactive area (*n* = 3; mean value taken from three separate microscopic fields). The data are presented as mean ± SEM, and statistical significance was tested using unpaired t-test between the two groups, followed by Bonferroni post-hoc corrections (*p < 0.05, **p < 0.01, ***p < 0.001).

For this study, 15-week-old WT male C57BL/6J mice were either fed with a normal chow diet (LEAN) or CD-HFD (MASLD) continuously for 20 weeks (Figure 1A). First, we wanted to confirm the MASLD phenotype and pathophysiology in the MASLD group of mice because of CD-HFD feeding. We observed that both the liver weight and the liver-to-body weight ratio were significantly increased in the MASLD group (Figure 1B, C; ***p < 0.001). Furthermore, we performed the analysis of various serum biomarkers associated with liver function and observed that the serum ALT (Figure 1D; ***p < 0.001), AST (Figure 1E; ***p < 0.001), hepcidin (Figure 1F; **p < 0.01), and ferritin (Figure 1G; *p < 0.05) levels were significantly elevated in the MASLD mice, indicating liver injury in these mice. Next, liver slices from both the LEAN and MASLD mouse groups were analyzed for histological changes by H&E staining and were assessed by the NAS in a blinded fashion. Our analysis showed that MASLD mice presented increased steatosis, lobular inflammation, and hepatocyte ballooning compared to the LEAN mouse group (Figure 1H, I). Furthermore, PSR staining was performed to analyze the overall fibrosis stage in the livers of these experimental mice. We detected mild perisinusoidal fibrosis in the livers of the MASLD mouse group (Figure 1H, I), with increased collagen deposition (Figure 1H, J; ***p < 0.001). In contrast, no fibrosis was found in the livers of the LEAN mouse group (Figure 1H, I). Furthermore, we performed immunohistochemistry staining of *α*-SMA and IL-1β using the liver slices. Our result showed that both *α*-SMA (Figure 1H, K; ***p < 0.001) and IL-1β (Figure 1H, L; ***p < 0.001) expression were significantly increased in the MASLD group compared to the LEAN mice, indicating clear hepatic inflammation and activation of hepatic stellate cells due to CD-HFD feeding for 20 weeks.

### 3.2. Mice with underlying MASLD-like phenotype are more susceptible to non-cholera vibriosis infection compared to their LEAN counterparts

**Figure 2.**
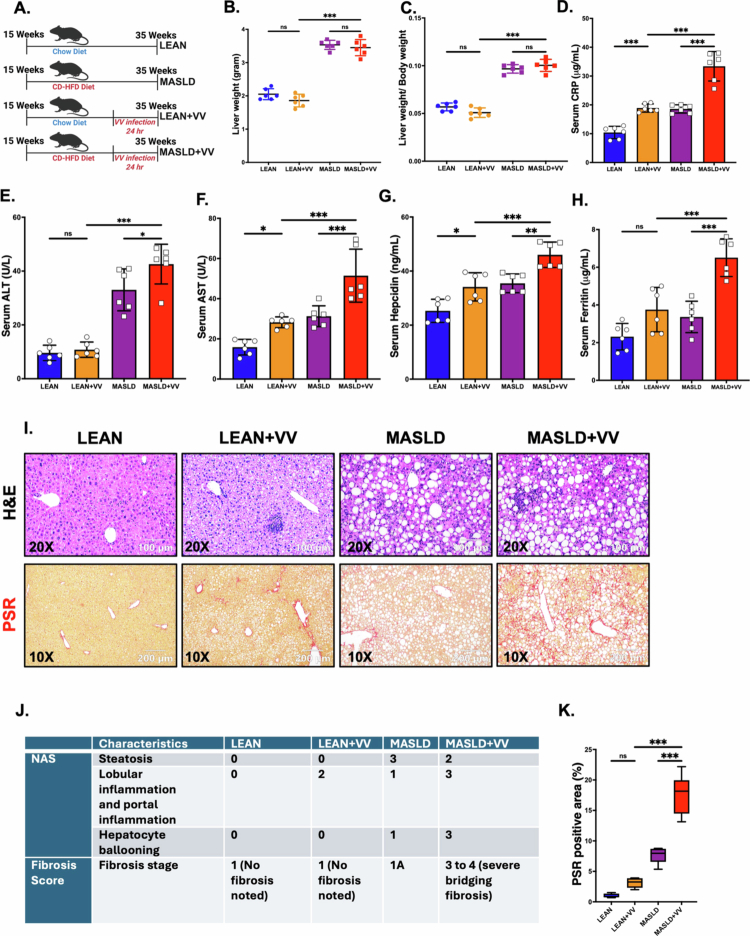
Underlying MASLD conditions in mice caused increased non-cholera vibriosis and affected the hepatic pathophysiology. (A) Schematic representation of the experimental mouse groups: LEAN [*n* = 6; chow diet-fed mice for 20 weeks], LEAN + VV [*n* = 6; mice fed with chow diet for 20 weeks and received oral VV inoculation for 24 h], MASLD [*n* = 6; CD-HFD fed mice for 20 weeks], and MASLD + VV [*n* = 6; mice fed with CD-HFD for 20 weeks and received oral VV inoculation for 24 h]. (B) Liver weight (grams), and (C) liver-to-body weight ratio of the LEAN, LEAN + VV, MASLD, and MASLD + VV mouse groups. Serum levels of (D) CRP (μg/mL), (E) ALT (U/L), (F) AST (U/L), (G) hepcidin (ng/mL), and (H) ferritin (μg/mL) in the LEAN, LEAN + VV, MASLD, and MASLD + VV mouse groups. Formalin-fixed, paraffin-embedded 5 μm liver slices from the LEAN, LEAN + VV, MASLD, and MASLD + VV mouse groups were used for histopathological analyses. Representative images of (I) hematoxylin and eosin (H&E) staining and Picrosirius red (PSR) staining of liver sections of LEAN, LEAN + VV, MASLD, and MASLD + VV mouse groups. H&E images were captured at 200× magnification, whereas PSR images were captured at 100× magnification. (J) NAFLD activity score (NAS) and fibrosis score for the LEAN, LEAN + VV, MASLD, and MASLD + VV mouse groups. (K) Morphometric analyses (calculated as %ROI) of PSR staining, where the Y-axis represents % positive immunoreactive area (*n* = 3; mean value taken from three separate microscopic fields). Data were represented as mean ± SEM, and statistical significance was tested using one-way ANOVA between all the groups, followed by Bonferroni post-hoc corrections (ns = non-significant, *p < 0.05, **p < 0.01, ***p < 0.001).

Next, we wanted to assess whether the pre-existing underlying MASLD-like phenotype in mice had any effect on the non-cholera vibriosis susceptibility. For that purpose, we infected both 35-week-old chow diet-fed LEAN mice (LEAN + VV) and CD-HFD diet-fed MASLD mice (MASLD + VV) with intragastric VV inoculum for 24 hours (Figure 2A). qRT-PCR of the *vvhA* gene was performed to confirm the VV infection using the feces of the mice (Supplementary Figure S1). Although CD-HFD feeding in the MASLD cohort of mice increased the liver weight and liver-to-body weight ratio, our results showed that VV infection alone did not affect either the liver weight or the liver-to-body weight ratio (Figure 2B, C; ns, ns, respectively). However, oral administration of VV significantly increased the serum CRP levels (μg/mL) in the LEAN + VV group compared to the LEAN group (Figure 2D; ***p < 0.001) and the MASLD + VV group compared to the MASLD group (Figure 2D; ***p < 0.001) as detected by ELISA. Furthermore, we also found that the serum CRP level in the MASLD + VV mice was significantly elevated compared to the LEAN + VV group (Figure 2D; ***p < 0.001). These results confirmed the VV-mediated onset of infection in these experimental mice.

Next, we analyzed the serum biomarkers associated with liver injury. Our results detected that the MASLD + VV mice had significantly elevated levels of serum ALT (Figure 2E; ***p < 0.001, **p < 0.05, respectively), AST (Figure 2F; ***p < 0.001, ***p < 0.001, respectively), hepcidin (Figure 2G; ***p < 0.001, **p < 0.01, respectively), and ferritin (Figure 2H; ***p < 0.001, ***p < 0.001, respectively) compared to either the LEAN + VV or the MASLD groups. In contrast, VV infection in LEAN mice significantly increased the serum AST (Figure 2F; *p < 0.05) and hepcidin (Figure 2G; *p < 0.05) levels but did not significantly increase the serum ALT (Figure 2E; ns), and Ferritin levels (Figure 2H; ns).

We also analyzed liver slices from these mouse groups for histological changes by H&E staining and assessed them by NAS in a blinded manner. Our analysis revealed that MASLD + VV mice presented increased steatosis, severe lobular inflammation, and hepatocyte ballooning compared to the other mouse groups (Figure 2I, J). Moreover, the PSR staining results detected significantly higher levels of collagen deposition (Figure 2I, K; ***p < 0.001) and severe bridging fibrosis with cirrhotic lesions in the livers of the MASLD + VV mice (Figure 2I, J) compared to either the LEAN + VV group or only the MASLD group. In contrast, oral VV inoculation in LEAN mice resulted in increased lobular and portal inflammation compared to the LEAN group, but no fibrosis was noted (Figure 2I-K; ns). These results showed that underlying MASLD conditions in mice could play a key role in increasing susceptibility to gut pathogens such as VV.

**Figure 3.**
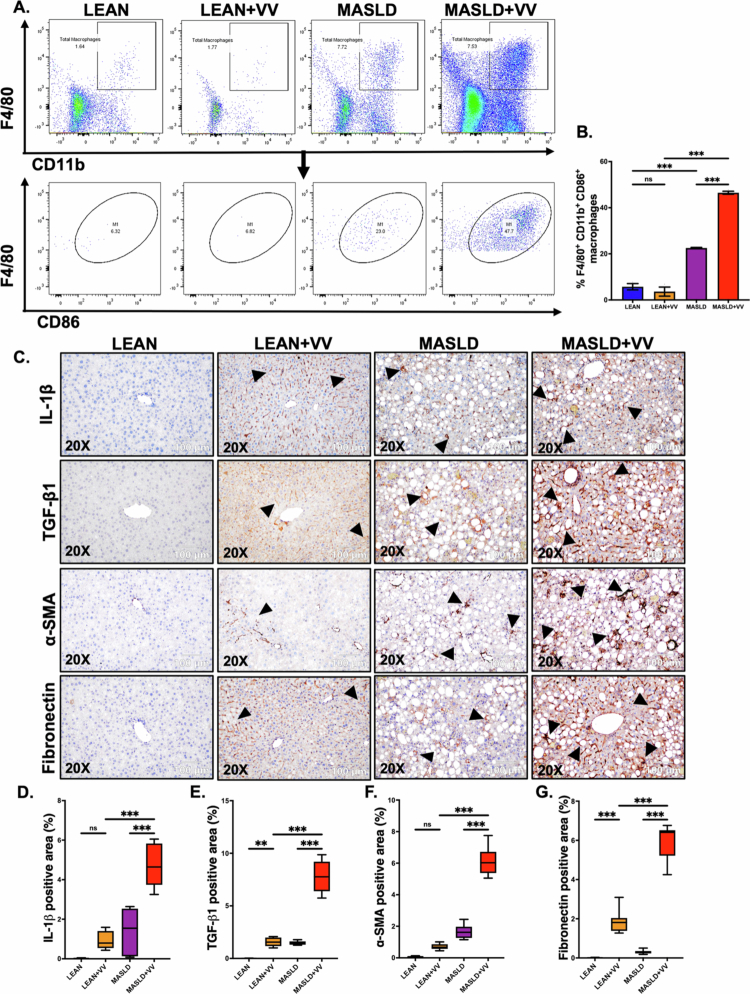
Underlying MASLD conditions in mice caused increased M1 macrophage polarization and inflammation after oral VV inoculation, leading to hepatic fibrosis. (A, B) Frequencies of the M1 macrophage (CD45^+^F4/80^+^CD11b^+^CD86^+^) population (represented by %) in the livers from the LEAN, LEAN + VV, MASLD, and MASLD + VV mouse groups. Formalin-fixed, paraffin-embedded 5 μm liver slices from the LEAN, LEAN + VV, MASLD, and MASLD + VV mouse groups were used for histopathological analyses. (C) Representative immunohistochemistry images depicting IL-1β, TGF-β1, *α*-SMA, and fibronectin immunoreactivity (indicated by black arrowheads) in the liver sections of the LEAN, LEAN + VV, MASLD, and MASLD + VV mouse groups. All the immunohistochemistry images were captured at 200× magnification. Morphometric analyses (calculated as %ROI) of (D) IL-1β, (E) TGF-β1, (F) *α*-SMA, and (G) Fibronectin immunoreactivity, where the Y-axis represents the % positive immunoreactive area (*n* = 3; mean value taken from three separate microscopic fields). The data are presented as mean ± SEM, and statistical significance was tested using one-way ANOVA between all the groups, followed by Bonferroni post-hoc corrections (ns = non-significant, *p < 0.05, **p < 0.01, ***p < 0.001).

Next, we wanted to assess M1 macrophage-mediated hepatic inflammation in these experimental mouse groups. For that purpose, single-cell NPC suspensions were prepared from the mice’s livers, and flow cytometry was performed to detect the M1 macrophage (CD45^+^F4/80^+^CD11b^+^CD86^+^) population (gating strategy is outlined in Supplementary Figure S4). We detected a significant increase in the population of M1 macrophages in the MASLD + VV group compared to either the MASLD only or the LEAN + VV groups (Figure 3A, B; ***p < 0.001, ***p < 0.001, respectively). We next wanted to detect the hepatic expression of IL-1β, a pro-inflammatory cytokine released by M1 macrophages, in these mouse groups. For that purpose, immunohistochemistry was performed on liver slices. Our result showed that IL-1β expression was significantly elevated in the hepatic sinusoids of MASLD + VV mice compared to either the LEAN + VV group or the MASLD-only group (Figure 3C, D; ***p < 0.001, ***p < 0.001, respectively). However, we did not observe significantly increased IL-1β expression in the LEAN + VV mice compared to the control LEAN group (Figure 3C, D; ns).

We also wanted to analyze whether intragastric VV inoculation in MASLD mice had any effect on the TGF-β1-mediated hepatic stellate cell activation and the resulting extracellular matrix protein deposition in the liver. Immunohistochemistry images of the liver sections revealed that VV inoculation in underlying MASLD conditions triggered the expression of TGF-β1 significantly, resulting in increased hepatic stellate cell activation (marked by *α*-SMA expression) and fibronectin deposition in the MASLD + VV group compared to the LEAN + VV group or MASLD mice (Figure 3C-G; ***p < 0.001). In contrast, VV infection in LEAN mice significantly increased TGF-β1 (Figure 3C, E; **p < 0.01) and fibronectin expression (Figure 3C, G; ***p < 0.001) levels, but *α*-SMA expression was not significantly elevated (Figure 3C, F; ns). These results clearly indicated that MASLD mice had a more severe form of non-cholera vibriosis compared to the LEAN mice, indicating that underlying liver disease conditions can play a key role in disease severity.

### 3.3. Mice with underlying MASLD-like phenotype possess an altered intestinal microenvironment

**Figure 4.**
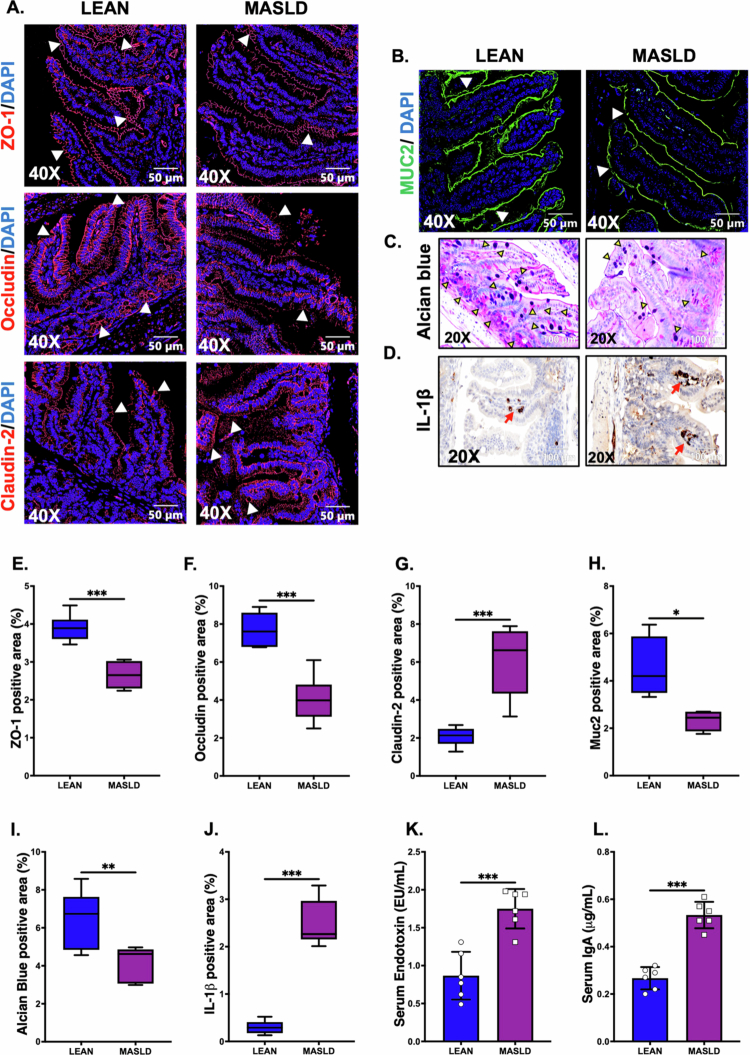
Underlying MASLD conditions in mice led to altered intestinal homeostasis. Representative immunofluorescence images depicting (A) ZO-1, Occludin, and Claudin-2 immunoreactivity (red and indicated by white arrowheads), (B) MUC2 immunoreactivity (green and indicated by white arrowheads) in the small intestine sections of the LEAN and MASLD mouse groups. The sections were counterstained with DAPI (blue), and all the images were captured at 400× magnification. (C) Representative images of Alcian Blue PAS staining and (D) immunohistochemistry images depicting IL-1β immunoreactivity (indicated by red arrows) in the small intestine sections of LEAN and MASLD mouse groups. The images were captured at 200× magnification. Morphometric analyses (calculated as %ROI) of (E) ZO-1, (F) Occludin, and (G) Claudin-2, (H) MUC2 immunoreactivity (I) Alcian Blue PAS staining, and (J) IL-1β immunoreactivity, where the Y-axis represents the % positive immunoreactive area (*n* = 3; mean value taken from three separate microscopic fields) (*p* = 0.003, *p* < 0.001, *p* = 0.003, respectively). Serum levels of (K) endotoxin (EU/mL) and (L) IgA (μg/mL) in the LEAN and MASLD mouse groups. The data are presented as mean ± SEM, and statistical significance was tested using unpaired t-test between the two groups, followed by Bonferroni post-hoc corrections (*p < 0.05, **p < 0.01, ***p < 0.001).

As the VV inoculum was administered via oral gavage, we hypothesized that an altered or disrupted intestinal epithelial barrier due to underlying MASLD conditions could potentiate the risk of increased VV release in the bloodstream via portal circulation, which directly connects the gut to the liver. Following this rationale, we wanted to examine the expression of different intestinal tight junction (TJ) proteins that are responsible for intestinal epithelial barrier integrity. Immunofluorescence staining of different TJ proteins, including ZO-1, Occludin, and Claudin-2, was performed using the small intestine tissue sections from the LEAN and MASLD groups. Our results showed a decreased expression of the ZO-1 and Occludin proteins and a parallel increase in Claudin-2 expression in the intestinal sections of the MASLD group compared to the LEAN mice (Figure 4A, E‒G; ***p < 0.001, ***p < 0.001, ***p < 0.001, respectively). A disrupted or leaky intestinal barrier can lead to increased release of gut-derived endotoxins in the systemic circulation. Therefore, an LAL assay was conducted to detect endotoxin levels in the serum samples of these experimental mouse groups. We detected significantly elevated endotoxemia in the MASLD mouse group compared to the LEAN mice group (Figure 4K; ***p < 0.001), thereby confirming our findings about intestinal barrier dysfunction due to altered TJ protein expression.

In addition to the intestinal epithelial barrier, the mucus layer that covers the villi also plays a pivotal role in protecting the host from invading pathogens and regulating the passage of water, ions, and various immune mediators, e.g., antimicrobial peptides and immunoglobulin A (IgA) in the lumen. Mucin 2 (MUC2) is the key protein associated with this protective mucus layer and is produced by the goblet cells residing in the intestinal epithelia.^30^ In our study, we wanted to examine whether MASLD conditions induced by CD-HFD feeding had any effect on mucus layer thickness. Immunofluorescence staining depicted that MUC2 expression was significantly lower in the MASLD mouse group compared to the LEAN group (Figure 4B, H; *p < 0.05), indicating possible thinning of the intestinal mucus layer due to the underlying MASLD-like phenotype. Furthermore, we also performed Alcian blue periodic acid–Schiff (PAS) staining to detect mucin-producing goblet cells in the intestinal epithelia. Our results showed that the number of mucin-producing goblet cells was lower in the MASLD mouse group compared to the LEAN group (Figure 4C, I; **p < 0.01), thereby corroborating our previous finding of decreased mucus layer thickness.

Next, we wanted to investigate intestinal inflammation, as the underlying MASLD-like phenotype is often associated with chronic, low-grade inflammation. Our immunohistochemistry results clearly showed that the expression of the pro-inflammatory cytokine IL-1β was significantly increased in the intestinal sections of MASLD mice compared to the LEAN group (Figure 4D, J; ***p < 0.001). Finally, the serum IgA levels in these experimental mouse groups were also quantified by ELISA, and we detected a significant increase in IgA in the MASLD mouse group compared to the LEAN mice (Figure 4L; ***p < 0.001).

### 3.4. Perturbation of the intestinal microenvironment augments severe non-cholera vibriosis infection in mice with underlying MASLD-like phenotype

**Figure 5.**
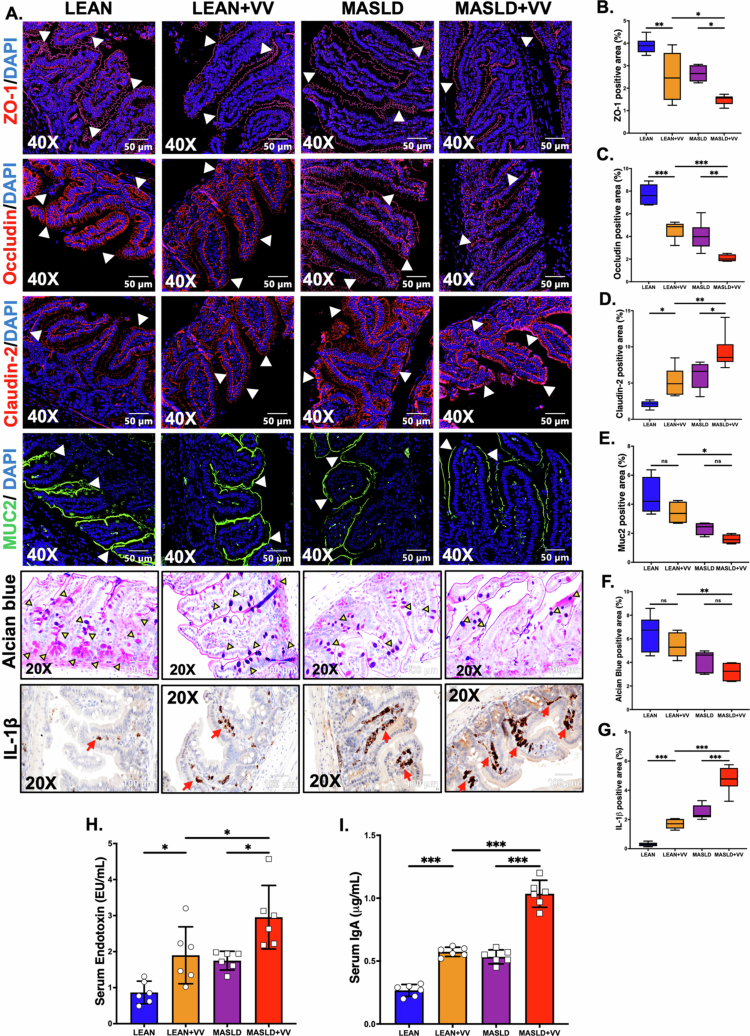
An altered intestinal microenvironment under MASLD conditions exacerbates increased non-cholera vibriosis in mice. (A) Representative immunofluorescence images depicting ZO-1, Occludin, Claudin-2 (red), MUC2 (green) immunoreactivity (indicated by white arrowheads), Alcian Blue PAS staining, and immunohistochemistry images depicting IL-1β immunoreactivity (indicated by red arrows) in the small intestine sections of LEAN, LEAN + VV, MASLD, and MASLD + VV mouse groups. For immunofluorescence, the sections were counterstained with DAPI (blue), and all the images were captured at 400× magnification. For Alcian Blue PAS staining and immunohistochemistry, all the images were captured at 200× magnification. (C) Representative images of. The images were captured at 200× magnification. Morphometric analyses (calculated as %ROI) of (B) ZO-1, (C) Occludin, (D) Claudin-2, (E) MUC2 immunoreactivity (F) Alcian Blue PAS staining, and (G) IL-1β immunoreactivity, where the Y-axis represents % positive immunoreactive area (*n* = 3; mean value taken from three separate microscopic fields) (*p* = 0.003, *p* < 0.001, *p* = 0.003, respectively). The serum levels of (H) endotoxin (EU/mL) and (l) IgA (μg/mL) in the LEAN, LEAN + VV, MASLD, and MASLD + VV mouse groups. The data are presented as mean ± SEM, and statistical significance was tested using unpaired t-test between the two groups, followed by Bonferroni post-hoc corrections (ns = non-significant, *p < 0.05, **p < 0.01, ***p < 0.001).

As we detected significant alterations in intestinal homeostasis in the MASLD mouse groups, we sought to investigate the effects of intragastric VV inoculation in these mice with pre-existing liver disease conditions. Immunofluorescence staining was conducted to observe changes in TJ protein expression levels. Our results showed that oral VV administration in mice with underlying MASLD led to significantly decreased ZO-1 and Occludin expression compared to the LEAN + VV group (Figure 5A, B-C; *p < 0.05, ***p < 0.001, respectively) or MASLD-only mice (Figure 5A, B-C; *p < 0.05, **p < 0.01, respectively). In contrast, Claudin-2 expression was found to be significantly elevated in the MASLD + VV group compared to either the LEAN + VV (Figure 5A, D; **p < 0.01) group or the MASLD group (Figure 5A, D; *p < 0.05). These findings were further corroborated by the serum endotoxin levels detected by the LAL assay. We found that oral VV inoculation in both LEAN and MASLD mice resulted in increased endotoxemia compared to LEAN-only or MASLD-only groups (Figure 5H; *p < 0.05, *p < 0.05, respectively). Furthermore, the serum endotoxin level in the MASLD + VV mice was significantly higher compared to LEAN + VV mice (Figure 5H; *p < 0.05).

Next, we studied MUC2 expression and observed the presence of mucin-producing goblet cells in these experimental mouse groups by performing immunofluorescence and alcian blue PAS staining, respectively. Our results revealed that oral VV inoculation alone did not significantly influence MUC2 expression or the number of goblet cells in the epithelia, irrespective of LEAN or MASLD conditions (Figure 5A, E-F; ns).

However, intragastric VV inoculation triggered increased gut inflammation in both the LEAN + VV group and the MASLD + VV group compared to their respective control groups (Figure 5A, G; ***p < 0.001), as detected by IL-1β expression in the villi. In particular, IL-1β immunoreactivity was significantly greater in the MASLD + VV group compared to the LEAN + VV group (Figure 5A, G; ***p < 0.001). Consequently, serum IgA levels were also quantified by ELISA, and we observed a significant increase in systemic IgA in MASLD + VV mice compared to either the LEAN + VV group or the MASLD group (Figure 5I; ***p < 0.001). In addition, VV administration in LEAN mice also resulted in higher circulatory IgA levels compared to LEAN control mice (Figure 5I; ***p < 0.001).

### 3.5. Mice with underlying MASLD-like phenotype have altered gut microbiome and resistome patterns

In addition to the intestinal epithelial barrier and protective mucus layer, the gut microbiome is a key factor that shapes the host’s intestinal homeostasis, immune responses, and overall health. In our study, we also wanted to determine whether 20-week CD-HFD feeding had any effect on the gut microbiome profile of these mice, which already exhibited significant changes in the intestinal milieu.

**Figure 6.**
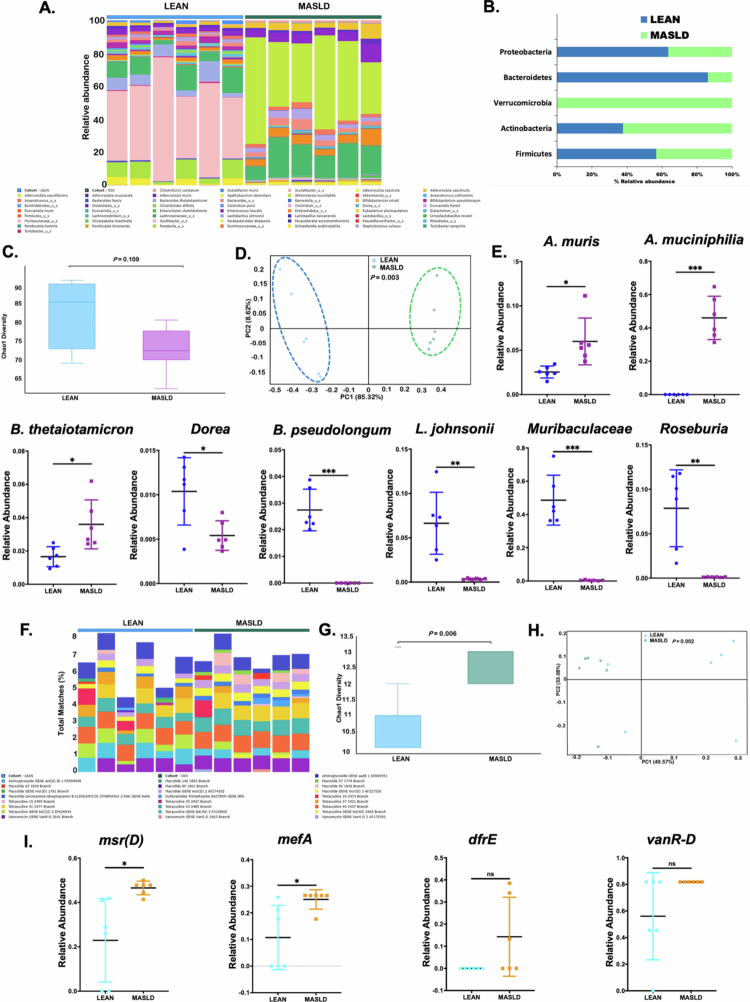
Mice with underlying MASLD conditions have separate gut microbiome and resistome patterns. (A) Heat map representing the overall gut bacteriome profile in the LEAN and MASLD groups. (B) The % relative abundance of the gut bacteriome at the phylum level. (C) Box plot depicting *α*-diversity (Shannon diversity) in the experimental groups (*p* = 0.873). (D) Bray–Curtis *β*-diversity plot for the gut bacteriome profile (*p* = 0.003). (E) The relative abundance of the individual bacterial species in the LEAN and MASLD groups. (F) Heat map representing the overall gut resistome profile in the LEAN and MASLD groups. (G) Box plot depicting *α*-diversity (Chao1 diversity) in the experimental groups (*p* = 0.006). (H) Bray–Curtis *β*-diversity plot for the gut bacteriome profile (*p* = 0.002). (I) The relative abundance of the individual ARGs in the LEAN and MASLD groups. Statistical significance was tested using the Wilcoxon rank sum test.

Next-generation sequencing was performed on the fecal pellets obtained from the LEAN and MASLD groups. The heatmap revealed a distinct cluster of bacteriome profiles for the LEAN group compared to the MASLD group (Figure 6A). At the phylum level, we observed an increased relative abundance of the phyla Verrucomicrobia and Actinobacteria with a parallel decreased abundance of the phyla Bacteroidetes, Firmicutes, and Proteobacteria in the MASLD group compared to the LEAN group (Figure 6B). Next, the overall changes in the gut microbiome composition between the two experimental mouse groups were estimated by diversity indices. *The α* diversity indices, represented by the Chao1 diversity index (Figure 6C; *p* = 0.109), Simpson diversity index (Supplementary Figure S2B; *p* = 0.873), and Shannon diversity index (Supplementary Figure S2C; *p* = 0.423), were marginally lower in the MASLD group compared to the LEAN group. Interestingly, the *β*-diversity plot, represented by Bray–Curtis dissimilarity, revealed distinctly formed clusters for the LEAN group and MASLD group (Figure 6D; *p* = 0.003), indicating a significantly different microbial species-level composition between these two groups. We also performed a species-level analysis of the experimental groups of mice. Several important gut residents, such as *Adlercreutzia muris*, *Akkermasia muciniphilia* (known for intestinal mucin degradation), and *Bacteroides thetaiotaomicron* (known for breaking down complex polysaccharides into simpler carbohydrates), were significantly increased in MASLD mice compared to the LEAN mice (Figure 6E; *p < 0.05, **p < 0.01, ***p < 0.001). In contrast, several beneficial gut commensals like *Dorea_u_s*, *Bifidobacterium pseudolongum*, *Lactobacillus johnsonii*, *Muribaculaceae_u_s*, and *Roseburia_u_s* were decreased in abundance in the CD-HFD fed MASLD mice compared to Chow diet-fed LEAN mice (Figure 6E; *p < 0.05, **p < 0.01, ***p < 0.001). Furthermore, the linear discriminant analysis effect size (LEfSe) bar chart clearly distinguished the microbiome signatures of the LEAN and MASLD mouse groups (Supplementary Figure S2A). Additionally, a decrease in the butyrogenic bacterial abundance in the gut was further confirmed by measuring fecal butyrate levels (ng/mL per mg of stool), where we observed a decrease in the fecal butyrate levels in the MASLD mouse group compared to the LEAN group (Supplementary Figure S2D).

As we observed a distinctively different gut microbiome profile in the MASLD mice, we also wanted to examine whether underlying MASLD conditions can lead to any changes in the gut resistome signature. A total of 25 ARGs were detected and found to be present in both the LEAN and MASLD groups, as represented by the heatmap (Figure 6F) and LEfSe bar chart (Supplementary Figure S2E). We also observed that the *α* diversity (marked by the Chao1 diversity index) of the gut resistome of the mice in the MASLD group was significantly greater (Figure 6G; **p < 0.01) compared to the LEAN mice. However, the Simpson and Shannon diversity indices for the gut resistome marginally decreased (Supplementary Figure S2F-G). Consequently, the *β*-diversity plot (represented by Bray–Curtis dissimilarity) also clearly differed between the LEAN and MASLD mice (Figure 6H; *p* = 0.002). Furthermore, we analyzed the relative abundance of individual ARGs in the two experimental mouse groups. Compared with those in LEAN mice, the expression of macrolide antibiotic resistance genes such as *msr(D)* and *mefA* was significantly increased (Figure 6I; *p < 0.05, *p < 0.05, respectively) in the MASLD mouse group. In addition, trimethoprim-resistant *dfrE* ARGs and vancomycin-resistant *vanR-D* ARGs were also found to be marginally increased in the MASLD group compared to the LEAN group (Figure 6I; ns, ns, respectively).

### 3.6. Functional microbiome-host network highlights intestinal barrier-associated inflammatory and fibrotic states in MASLD in potentiating infection susceptibility

**Figure 7.**
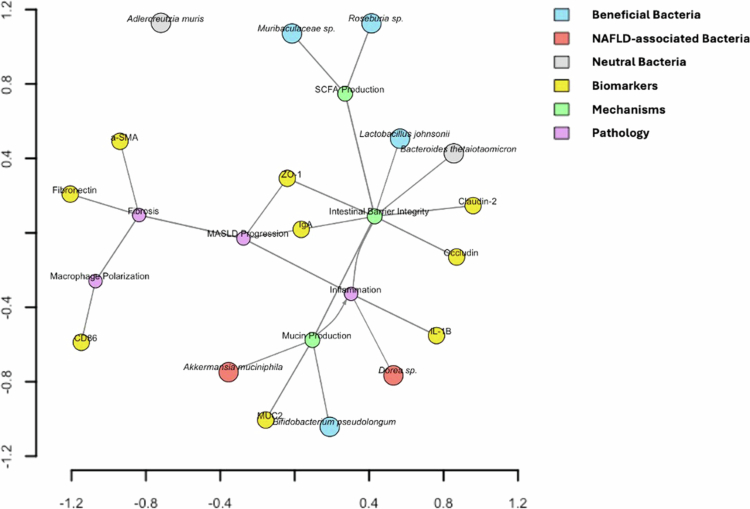
Functional microbiome‒host interaction network in MASLD. The network integrates gut microbial taxa with intestinal barrier functions, immune signaling, and downstream hepatic inflammatory and fibrotic outcomes relevant to MASLD. Nodes represent bacterial taxa, host barrier and immune markers, functional modules, and disease-related outcomes. Edge directions indicate biologically plausible relationships supported by prior experimental studies rather than inferred causality, and edge thickness reflects qualitative confidence based on literature support and concordance with observed patterns. Barrier-associated functions, including short-chain fatty acid production, mucin production, and epithelial integrity, are depicted as protective modules that constrain immune activation, while disruption of these processes is modeled as permissive for inflammation, macrophage activation, and fibrotic remodeling.

To place the observed microbiome alterations in MASLD into a broader biological context, we developed a functional microbiome–immune network that integrates disease- and health-associated taxa with established intestinal barrier, inflammatory, and hepatic fibrotic pathways (Figure 7). The network brings together bacterial taxa commonly reported in metabolic health or disease, including *Akkermansia muciniphila*, *Muribaculaceae* sp., *Roseburia* sp., *Bifidobacterium pseudolongum*, and *Lactobacillus johnsonii*, with host markers spanning epithelial barrier integrity, immune activation, macrophage polarization, and extracellular matrix remodeling. Taxa typically linked to metabolic resilience are positioned proximal to functional modules related to short-chain fatty acid production and mucin biology. These modules are connected to markers of epithelial barrier integrity, e.g., ZO-1, Occludin, Claudin-2, and IgA. This organization reflects prior experimental evidence showing that SCFAs, particularly butyrate, support epithelial barrier function and tight junction assembly^31^ and that SCFAs more broadly regulate intestinal homeostasis and immune tone.^32^ In parallel, commensal-driven mucin production has been shown to maintain a structured mucus layer that physically separates luminal microbes from the intestinal epithelium, largely through MUC2-dependent mechanisms.^33^ In the network, these pathways are therefore represented as barrier-supportive processes that collectively limit immune activation rather than as direct drivers of downstream outcomes. In contrast, taxa associated with MASLD clustered closer to inflammatory and fibrotic nodes. This spatial organization is consistent with prior reports linking microbial dysbiosis to impaired barrier function and increased immune stimulation. Importantly, the network does not treat disruption of the mucus layer or barrier integrity as a direct cause of inflammation. Instead, reduced mucin production and weakened epithelial integrity are modeled as conditions that may permit greater epithelial exposure to microbial products, thereby increasing susceptibility to inflammatory signaling. This interpretation aligns with experimental studies demonstrating that loss of the mucus barrier facilitates bacterial contact with the epithelium and enhances immune engagement, while sustained inflammation often requires additional contributing factors.^34^^,^^35^ Downstream inflammatory markers, including IL-1β, are linked to macrophage activation and fibrotic readouts such as CD86, *α*-SMA, and fibronectin. These connections reflect well-established interactions between inflammatory signaling, macrophage polarization, and fibrogenic remodeling in metabolic liver disease. Previous studies have shown that inflammatory macrophages play a central role in hepatic stellate cell activation and extracellular matrix deposition during steatohepatitis and fibrosis.^36^ In addition, IL-1 signaling has been implicated in promoting the progression from steatosis to steatohepatitis and fibrotic pathology in experimental models, supporting the biological plausibility of the relationships represented in the network.^37^

Taken together, this functional network provides a literature-informed synthesis of host‒microbiome interactions relevant to MASLD. The directionality of edges reflects biologically plausible pathways supported by experimental evidence, while edge weights indicate relative confidence based on the strength of published data and concordance with observed patterns rather than inferred causality. As such, the network serves as a framework for generating testable hypotheses, including whether early impairments in SCFA production, mucin biology, or tight junction integrity precede inflammatory escalation and whether restoring these barrier-associated functions can mitigate macrophage activation and downstream fibrotic remodeling.

### 3.7. FMT in mice with an underlying MASLD-like phenotype showed decreased susceptibility to non-cholera vibriosis infection

As we detected that the mice with pre-existing liver disease conditions had increased susceptibility to non-cholera vibriosis, possibly due to disrupted intestinal homeostasis in combination with an altered gut microbiome and resistome profile, we further wanted to validate the role of the gut microbiome. Therefore, the ABX cocktail was first administered to the mice for 15 d to deplete the gut microbiome. Our results showed that ABX treatment did not affect the overall hepatic pathophysiology in either LEAN (LEAN + ABX) or MASLD (MASLD + ABX) mice, but significantly depleted the bacterial load in the gut (Supplementary Figure S3A-F).

**Figure 8.**
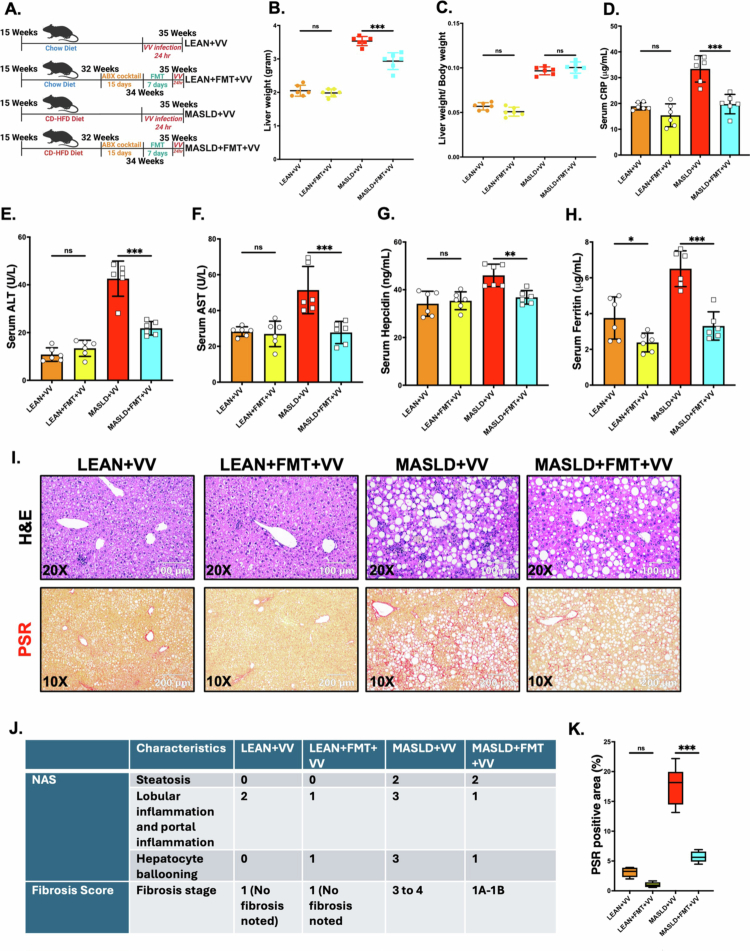
FMT in mice with underlying MASLD conditions showed improved pathophysiological outcomes Compared to non-cholera vibriosis infection. (A) Schematic representation of the experimental mouse groups: LEAN + VV [*n* = 6; mice fed with chow diet for 20 weeks and received oral VV inoculation for 24 h], LEAN + FMT + VV [*n* = 6; chow diet-fed mice, first treated with the ABX cocktail for 15 d followed by FMT for 7 d, and received oral VV inoculation for 24 h], MASLD + VV [*n* = 6; mice fed with CD-HFD for 20 weeks and received oral VV inoculation for 24 h], and MASLD + FMT + VV [*n* = 6; CD-HFD fed mice, first treated with the ABX cocktail for 15 d followed by FMT for 7 d, and received oral VV inoculation for 24 h]. (B) Liver weight (grams), and (C) liver-to-body weight ratio of the LEAN + VV, LEAN + FMT + VV, MASLD + VV, and MASLD + FMT + VV mouse groups. Serum levels of (D) CRP (μg/mL), (E) ALT (U/L), (F) AST (U/L), (G) Hepcidin (ng/mL), (H) Ferritin (μg/mL) in the LEAN + VV, LEAN + FMT + VV, MASLD + VV, and MASLD + FMT + VV mouse groups. Formalin-fixed, paraffin-embedded 5 μm liver slices were used for histopathological analyses. Representative images of (I) hematoxylin and eosin (H&E) staining and picrosirius red (PSR) staining in the liver sections from the LEAN + VV, LEAN + FMT + VV, MASLD + VV, and MASLD + FMT + VV mouse groups. H&E images were captured at 200× magnification, whereas PSR images were captured at 100× magnification. (J) NAFLD activity score (NAS) and fibrosis score for the LEAN + VV, LEAN + FMT + VV, MASLD + VV, and MASLD + FMT + VV mouse groups. (K) Morphometric analyses (calculated as %ROI) of PSR staining, where the Y-axis represents % positive immunoreactive area (*n* = 3; mean value taken from three separate microscopic fields). The data are presented as mean ± SEM, and statistical significance was tested using one-way ANOVA between all the groups, followed by Bonferroni post-hoc corrections (ns = non-significant, *p < 0.05, **p < 0.01, ***p < 0.001).

Next, we performed FMT in both the ABX-pretreated LEAN (LEAN + FMT + VV) and MASLD (MASLD + FMT + VV) mouse groups for 7 d, followed by intragastric VV inoculum treatment for 24 h (Figure 8A). We observed that MASLD mice receiving FMT had significantly decreased liver weight compared to MASLD mice post-VV infection, but the liver-to-body weight ratio did not differ (Figure 8B, C; ***p < 0.001, ns, respectively). Similarly, the liver weight and liver-to-body weight ratio also showed no significant alterations between the LEAN + VV and LEAN + FMT + VV mouse groups (Figure 8B, C; ns, ns, respectively). However, MASLD mice that received FMT presented significantly lower serum CRP, ALT, AST, hepcidin, and ferritin levels compared to the MASLD mice post oral VV inoculation (Figure 8D–H; **p < 0.01, ***p < 0.001). In contrast, we observed only a slight decrease in the serum ferritin level between the LEAN + VV and LEAN + FMT + VV groups (Figure 8H; *p < 0.05).

Furthermore, the liver slices from these mouse groups were also analyzed for histological changes by H&E staining and were assessed by NAS. Our analysis showed that MASLD + FMT + VV mice had similar steatosis grades but lower levels of lobular and portal inflammation as well as hepatocyte ballooning compared to the MASLD + VV mice (Figure 8I, J). In contrast, livers from the LEAN + FMT + VV mice showed decreased levels of lobular and portal inflammation, but increased hepatocyte ballooning compared to the LEAN + VV group (Figure 8I, J). PSR staining results detected significantly decreased collagen deposition (Figure 8I, K; ***p < 0.001) and fibrosis (Figure 8I, J) in the livers of the MASLD + FMT + VV mice compared to the MASLD + VV group. In contrast, no fibrosis was detected in the livers of the LEAN + FMT + VV or the LEAN + VV mouse groups (Figure 8I, J).

**Figure 9.**
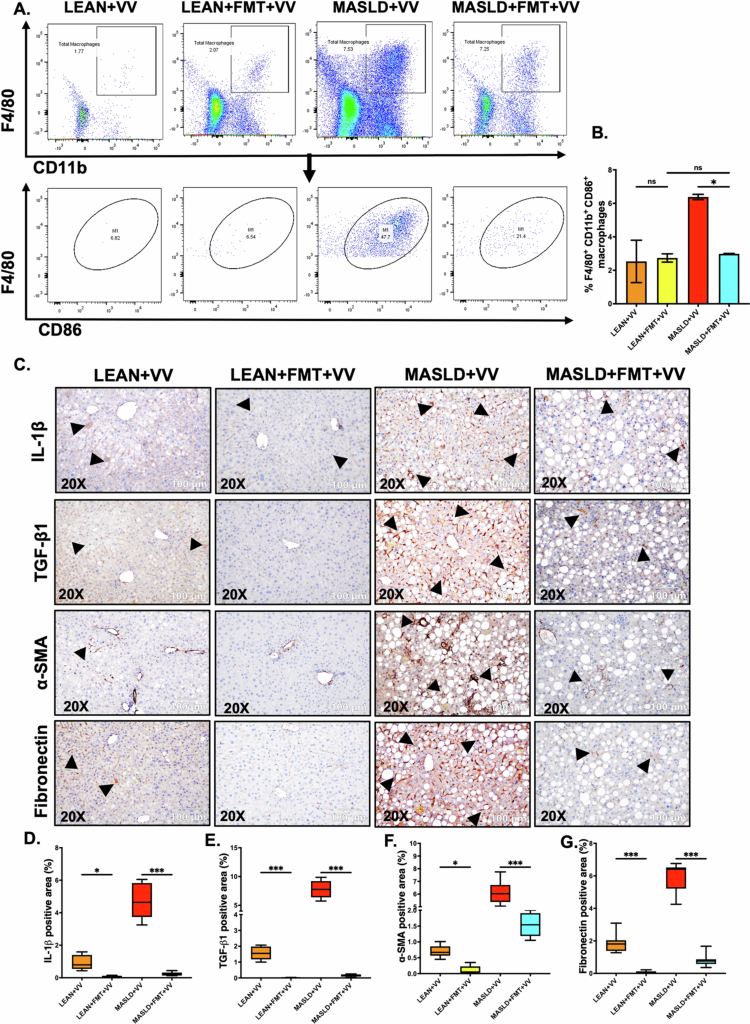
FMT in mice with underlying MASLD conditions showed decreased M1 macrophage polarization, inflammation, and fibrosis in the liver post-oral VV inoculation. (A, B) Frequencies of M1 macrophage (CD45^+^F4/80^+^CD11b^+^CD86^+^) population (represented by %) in the livers from the LEAN + VV, LEAN + FMT + VV, MASLD + VV, and MASLD + FMT + VV mouse groups. Formalin-fixed, paraffin-embedded 5 μm liver slices from the LEAN + VV, LEAN + FMT + VV, MASLD + VV, and MASLD + FMT + VV mouse groups were used for histopathological analyses. (C) Representative immunohistochemistry images depicting IL-1β, TGF-β1, *α*-SMA, and fibronectin immunoreactivity (indicated by black arrowheads) in the liver sections of the LEAN + VV, LEAN + FMT + VV, MASLD + VV, and MASLD + FMT + VV mouse groups. All the immunohistochemistry images were captured at 200× magnification. Morphometric analyses (calculated as %ROI) of (D) IL-1β, (E) TGF-β1, (F) *α*-SMA, and (G) Fibronectin immunoreactivity, where the Y-axis represents % positive immunoreactive area (*n* = 3; mean value taken from three separate microscopic fields). Data are presented as mean ± SEM, and statistical significance was tested using one-way ANOVA between all the groups, followed by Bonferroni post-hoc corrections (ns = non-significant, *p < 0.05, **p < 0.01, ***p < 0.001).

Flow cytometry analysis was also performed on NPCs isolated from the livers of these experimental groups. We detected a significant decrease in the CD86^+^ M1 macrophage population in the MASLD + FMT + VV group compared to the MASLD + VV group (Figure 9A, B; *p < 0.05). However, we also noted that there was no significant difference in CD86^+^ M1 macrophages between the LEAN + FMT + VV group compared to the LEAN + VV group (Figure 9A, B; ns). The immunohistochemistry results showed that IL-1β expression was significantly lower in the hepatic sinusoids of MASLD + FMT + VV mice compared to the MASLD + VV group, and LEAN + FMT + VV mice compared to the LEAN + VV group (Figure 9C, D; ***p < 0.001).

We also examined the markers of TGF-β1-mediated hepatic stellate cell activation and extracellular matrix protein deposition in the livers of these mouse groups. Immunohistochemistry images of liver sections showed that MASLD mice subjected to FMT presented significantly lower expression of TGF-β1 (Figure 9C, E; ***p < 0.001), *α*-SMA (Figure 9C, F; ***p < 0.001), and fibronectin (Figure 9C, G; ***p < 0.001) compared to the MASLD mice post VV inoculation. In addition, we also observed significant changes in TGF-β1 and *α*-SMA expression levels (Figure 9C, E-G; ***p < 0.001, *p < 0.05) between the LEAN + FMT + VV and LEAN + VV mouse groups.

These results validated that depletion of the gut microbiome followed by recolonization with a healthy microbiome by FMT protected the mice with underlying MASLD from the increased severity of non-cholera vibriosis and emphasized the importance of the altered microbiome’s role in these mice.

## 4. Discussion

An underlying MASLD-like phenotype had far worse outcomes in *Vibrio vulnificus*-challenged mice, primarily from altered gut dysbiosis and resistome patterns. Furthermore, the prevalence of low-grade intestinal inflammation in MASLD contributes to the rapid progression of MASLD to MASH, thereby confirming a second hit phenomenon. This is the first-ever mechanistic study that outlines a gut‒liver mechanistic axis involved in worsening outcomes of non-cholera vibriosis in MASLD.

In the present study, our results showed that feeding the mice a CD-HFD diet for 20 weeks significantly elevated levels of liver weight, liver-to-body weight ratio, serum ALT and AST levels, confirming liver injury. Furthermore, H&E images and NAS confirmed increased steatosis, hepatic inflammation, and mild fibrosis, which are hallmarks of steatohepatitis-like pathology. MASLD has previously been reported to be associated with dysfunctional intestinal homeostasis with increased gut leakiness, thinning of the protective mucus layer, and altered immune responses.^38^ Similar pathophysiological traits were also noted in these MASLD mice, including epithelial barrier dysfunction due to decreased TJ protein expression (ZO-1, Occludin), leading to increased endotoxemia, as well as a lower abundance of mucus-producing goblet cells in the intestinal epithelia and decreased expression of the MUC2 protein, indicating thinning of the protective mucus layer in the intestine. We also observed persistent and chronic gut inflammation and elevated levels of serum IgA in MASLD mice, indicating changes in the mucosal immune response. In addition, consistent with the clinical findings in patients with MASLD,^39^^,^^40^ elevated serum hepcidin and ferritin levels have also been detected in MASLD mice. Hepcidin is produced by hepatocytes and is known as the master iron regulator for iron absorption, storage, and circulation in the body.^41^ On the other hand, ferritin is an iron-binding protein that is mainly responsible for iron sequestration.^42^ Increased levels of both hepcidin and ferritin are found in patients with bacterial infections, as iron itself is a crucial element for bacterial pathogenesis.^43^ Considering these results, we deduced that these shifts in the underlying pathophysiological traits in MASLD mice might cause increased susceptibility to bacterial infections, as the gut and the liver have dynamic crosstalk via the “gut‒liver axis”.

Although cases of dermal exposure to VV are more prevalent, we aimed to use an intragastric route of VV administration in this study purely based on the rationale that foodborne cases of non-cholera vibriosis, particularly in patients with underlying liver disease conditions, have not been explored in detail. The pathogenesis of VV by oral inoculation was confirmed by the elevated serum CRP levels in the VV-treated LEAN and MASLD mice compared to their respective control groups. We detected that oral VV inoculation significantly damaged the livers of the MASLD mice, as high levels of circulatory ALT, AST, hepcidin, and ferritin were noted. More importantly, VV administration in mice also caused increased polarization of CD86^+^ M1 macrophages from total liver macrophages, leading to increased IL-1β release in the hepatic sinusoids, causing a high degree of lobular and portal inflammation. Furthermore, we noted that significant TGF-β1-mediated hepatic stellate cell activation ultimately caused severe extracellular matrix protein deposition and bridging fibrosis with some cirrhotic lesions in the livers of MASLD + VV mice. In contrast, VV inoculation in LEAN mice did not have the same degree of severity on the livers. As the dosage was administered orally, we assumed that the absorption and translocation of VV could be more challenging in the mice with underlying MASLD conditions because of increased gut permeability and thinning of the protective mucus layer. Our results also showed that VV administration in MASLD mice increased gut leakiness (decreased ZO-1 and Occludin expression with increased endotoxemia) even more significantly compared to either the LEAN + VV mice or the MASLD control group. However, the exact mechanism of the VV-mediated alteration of these TJ proteins is still unclear. In addition, we noted that VV inoculation caused increased acute gut inflammation and elevated serum IgA levels. These findings are also consistent with clinical reports since increased serum IgA is consistently present in patients with MASLD and serves as one of the major biomarkers of fibrosis.^44^ In contrast, we did not find any significant changes in MUC2 expression or the number of goblet cells in the VV-treated mouse groups compared to their respective controls. All these data plausibly imply that pre-existing mucus layer thinning and altered gut microbiome patterns in underlying MASLD conditions can enhance the likelihood of invasion by gut pathogens due to increased gut permeability.

In addition to the intestinal pathophysiology, MASLD also causes intestinal dysbiosis, as reported by several preclinical and clinical studies.^45-47^ However, the gut resistome signature in MASLD has not been reported before, leading us to investigate its potential role in increasing susceptibility to non-cholera vibriosis. The next-generation sequencing results showed that MASLD mice presented significantly altered overall gut microbiome composition (*β*-diversity by Bray–Curtis dissimilarity). Especially, species-level analysis showed a decreased abundance of several well-known probiotic gut bacteria, such as *Bifidobacterium pseudolongum*, which is known for improving gut immunity and anti-inflammatory effects,^48^ and *Lactobacillus johnsonii,* which is responsible for overall intestinal microflora balance, and intestinal barrier protection.^49^ Moreover, diet-induced MASLD conditions also decreased the abundance of *Muribaculaceae_u_s* and *Roseburia_u_s*, which are primarily responsible for propionate and butyrate production, respectively. Therefore, decreased production of these key SCFAs, especially butyrate, which we also identified in this study, could act as one of the key contributing factors to the pathophysiology. In parallel, we also noted an increased abundance of many gut residents due to CD-HFD feeding for 20 weeks. The abundance of *Akkermansia muciniphilia,* a beneficial gut commensal that can degrade glycoproteins of the intestinal mucus layer into simpler nutrients, was found to be increased in the MASLD group. Under normal physiological conditions, mucin degradation by this bacterium influences increased MUC2 expression by the host’s goblet cells to resist any invading gut pathogens, thereby maintaining dynamic equilibrium.^50^ However, a previous study by Desai et al. also showed that a decreased colonic mucus barrier due to fiber deprivation could lead to increased utilization of these mucus glycoproteins by *Akkermansia muciniphilia*, promoting susceptibility to enteric pathogens.^51^ In another study, the authors reported that over-colonization of *A. muciniphila* reduced the intestinal mucus layer and decreased the expression of TJ proteins,^52^ which also corroborated our findings. Furthermore, we also noted that the abundance of the prominent gut bacteria species *Bacteroides thetaiotaomicron* was increased in the mice with underlying MASLD. The exact role of *B. thetaiotaomicron* is still controversial, as it can serve as both a beneficial pathogen (breaking down polysaccharides)^53^ and an opportunistic pathogen^54^ in the host, depending on the normal/disease physiological conditions. Interestingly, in our earlier study, we detected that *B. thetaiotaomicron* could act as a reservoir for several ARGs, such as *mefA, msr(D)*, and *mel* in the intestine.^23^ Similarly, we also observed an increased relative abundance of the ARGs *msr(D)* and *mefA* in the MASLD group of mice, which could be harbored by *B. thetaiotaomicron* and could impart macrolide resistance. However, this is indeed speculative at this point, as the exact bacterial source for these ARGs has not been detected. In addition, we also observed increased diversity indices for ARGs in the MASLD group compared to the LEAN mice in resistome analysis. Combining our pathophysiological findings with bacteriome and resistome analysis clearly indicate that individuals with underlying MASLD conditions are likely to be more susceptible to GI infections and may possess a higher risk of antibiotic treatment failure owing to the increased accumulation of ARGs in the gut.

To further establish the role of the gut microbiome in underlying MASLD conditions, we utilized an ABX cocktail to deplete the gut microbiome and transplanted a “healthy” microbiome in both LEAN and MASLD mice, followed by oral VV inoculation. Our results clearly revealed that, compared with those in MASLD + VV mice, the recolonization of a healthy microbiome in these MASLD mice led to decreased severity of non-cholera vibriosis. Serum markers significantly improved ALT, AST, hepcidin, and ferritin levels. The liver histology results also showed lower levels of inflammation, ballooning, and significant improvement in fibrosis grade. However, we did not observe any significant difference in the overall percentage of CD86^+^ M1 macrophages, and overall liver inflammation was reduced, as indicated by the increase in IL-1β expression, which also supported our histological assessments by H&E staining. Interestingly, we also observed that gut-depleted LEAN mice with VV inoculation did not have a significant impact on the hepatic parameters compared to LEAN+VV mice, possibly due to other adaptive mechanisms. These results further emphasize gut microbiome-targeted therapeutic interventions (e.g., FMT) to ameliorate MASLD-mediated potential gastrointestinal complications.

Taken together, the findings of this study reveal the vital role of the intestinal microbiome and link it to intestinal physiological factors that could potentiate the increased risk of non-cholera vibriosis under MASLD conditions. Our mechanistic study also adds to the volume of epidemiological and clinical case reports that show the vulnerability of patients with MASLD to worsened outcomes in vibriosis. Furthermore, our mechanistic study will help establish clinical guidelines in non-cholera vibriosis as more cases have been identified in the United States and globally.

## 5. Limitations

Although our study identified the key underlying contributing factors related to the increased susceptibility of non-cholera vibriosis in the MASLD mice, the potential influence of other systemic factors related to MASLD (e.g., hepatic metabolic stress or weakened immunity) cannot be excluded completely. Histological analyses and subsequent scoring systems (NAS and fibrosis score) have also primarily validated in humans. Therefore, certain minute histological characteristics in patients with MASLD may not be fully reflected in CD-HFD diet-fed MASLD mice.^55^ Furthermore, our study primarily aimed to determine the acute effects of non-cholera vibriosis (24 h post-inoculation) that mimics the clinical scenarios in humans. Therefore, the long-term consequences of infection and the host’s immune response in these individuals with MASLD are still unknown, and this serves as a major limitation. Additionally, our study utilized metagenomic tools to identify only distinct species-level changes in the gut microbiome of MASLD mice. Therefore, the functional effects of these key species could not be evaluated precisely. Though the FMT experiment showed that transplantation with a 'healthy' gut microbiome can reverse the adverse phenotype, it is still difficult to pinpoint the microbiome’s role from other MASLD-related intestinal issues, e.g., pre-existing barrier dysfunction or chronic low-grade inflammation. Therefore, post-FMT improvements could reflect a synergistic effect of restoring microbial diversity, indirect repair of the intestinal wall, and a decreased inflammatory response. Furthermore, a complementary “reverse FMT” in healthy mice using stools from MASLD mice, followed by VV infection, may indicate whether the altered gut microbiome in MASLD alone could significantly potentiate the increased susceptibility to non-cholera vibriosis in the host. Consequently, though we have drawn a link between the observed increases in ARG and the higher risk of antibiotic treatment failure, we opine that our point is speculative and hypothetical at this point. The reason for this might be that there has been no effort so far to measure the antibiotic efficacy in preclinical models of non-cholera vibriosis. Our results indicate that the antibiotic efficacy of individual drugs tested (e.g., macrolides) can provide important evidence that can be used clinically.

## Supplementary Material

MASLD VV Supplementary File Revised.docx MASLD VV Supplementary File Revised.docx

## Data Availability

The data that support the findings of this study are available from the corresponding author, [SC], upon reasonable request. Access to the sequencing data is available via the Sequence Read Archive (SRA) database with accession ID PRJNA1348144.

## References

[1] Riazi K, Azhari H, Charette JH, Underwood FE, King JA, Afshar EE, Swain MG, Congly SE, Kaplan GG, Shaheen A. The prevalence and incidence of NAFLD worldwide: a systematic review and meta-analysis. Lancet Gastroenterol Hepatol. 2022;7(9):851–861. doi: 10.1016/S2468-1253(22)00165-0. 35798021

[2] Yki-Järvinen H. Non-alcoholic fatty liver disease as a cause and a consequence of metabolic syndrome. Lancet Diabetes Endocrinol. 2014;2(11):901–910. doi: 10.1016/S2213-8587(14)70032-4. 24731669

[3] Mantovani A, Morandin R, Fiorio V, Lando MG, Gaviraghi A, Motta L, Gobbi F, Tilg H, Byrne CD, Targher G. Association between MASLD and increased risk of serious bacterial infections requiring hospital admission: A meta-analysis. Liver Int. 2025;45(4):e16101. doi: 10.1111/liv.16101. 39258758 PMC11892334

[4] Patel J, Sohal A, Bains K, Chaudhry H, Kohli I, Khanna T, Dukovic D, Roytman M. Association of metabolic dysfunction-associated fatty liver disease with gastrointestinal infections: insights from National Inpatient Sample Database. BMJ Open Gastroenterol. 2024;11(1):e001224. doi: 10.1136/bmjgast-2023-001224. PMC10870785 38237944

[5] Benedé-Ubieto R, Cubero FJ, Nevzorova YA. Breaking the barriers: the role of gut homeostasis in Metabolic-Associated Steatotic Liver Disease (MASLD). Gut Microbes. 2024;16(1):2331460. doi: 10.1080/19490976.2024.2331460. 38512763 PMC10962615

[6] Trinanes J, Martinez-Urtaza J. Future scenarios of risk of Vibrio infections in a warming planet: a global mapping study. Lancet Planet Health. 2021;5(7):e426–e435. doi: 10.1016/S2542-5196(21)00169-8. 34245713

[7] Newton A, Kendall M, Vugia DJ, Henao OL, Mahon BE. Increasing rates of vibriosis in the United States, 1996-2010: review of surveillance data from 2 systems. Clin Infect Dis. 2012;54 Suppl 5(05):S391–5. doi: 10.1093/cid/cis243. 22572659 PMC4604744

[8] Mora C, McKenzie T, Gaw IM, Dean JM, von Hammerstein H, Knudson TA, Setter RO, Smith CZ, Webster KM, Patz JA, et al. Over half of known human pathogenic diseases can be aggravated by climate change. Nat Clim Chang. 2022;12(9):869–875. doi: 10.1038/s41558-022-01426-1. 35968032 PMC9362357

[9] Baker-Austin C, Trinanes J, Gonzalez-Escalona N, Martinez-Urtaza J. Non-Cholera Vibrios: The Microbial Barometer of Climate Change. Trends Microbiol. 2017;25(1):76–84. doi: 10.1016/j.tim.2016.09.008. 27843109

[10] Rippey SR. Infectious diseases associated with molluscan shellfish consumption. Clin Microbiol Rev. 1994;7(4):419–425. doi: 10.1128/CMR.7.4.419. 7834599 PMC358335

[11] Horseman MA, Surani S. A comprehensive review of Vibrio vulnificus: an important cause of severe sepsis and skin and soft-tissue infection. Int J Infect Dis. 2011;15(3):e157–66. doi: 10.1016/j.ijid.2010.11.003. 21177133

[12] Baker-Austin Craig, Oliver James D., Alam Munirul, Ali Afsar, Waldor Matthew K., Qadri Firdausi, Martinez-Urtaza Jaime, et al. Vibrio spp. infections. Nature Reviews Disease Primers. 2018;4(1):19. doi: 10.1038/s41572-018-0005-8. 30002421

[13] Haq SM, Dayal HH. Chronic liver disease and consumption of raw oysters: a potentially lethal combination--a review of Vibrio vulnificus septicemia. Am J Gastroenterol. 2005;100(5):1195–1199. doi: 10.1111/j.1572-0241.2005.40814.x. 15842598

[14] Christou L, Pappas G, Falagas ME. Bacterial infection-related morbidity and mortality in cirrhosis. Am J Gastroenterol. 2007;102(7):1510–1517. doi: 10.1111/j.1572-0241.2007.01286.x. 17509025

[15] Nazir S, Brown K, Shin AK, Donato AA. Vibrio vulnificus infection and liver cirrhosis: a potentially lethal combination. BMJ Case Rep. 2016;2016:bcr2016214772. doi: 10.1136/bcr-2016-214772. PMC4885366 27151052

[16] Aldiabat M, Kilani Y, Madi MY, Saha P, Roy S, Rockey DC, Chatterjee S, Syn WK, et al. Increased susceptibility to vibrio vulnificus infection in patients with MASLD, cirrhosis, and chronic liver diseases. Am J Med Sci. 2025;370(3):305–307. doi: 10.1016/j.amjms.2025.06.002. 40482969

[17] Lipinski JH, Zhou X, Gurczynski SJ, Erb-Downward JR, Dickson RP, Huffnagle GB, Moore BB, O’Dwyer DN, Raffatellu M. Cage Environment Regulates Gut Microbiota Independent of Toll-Like Receptors. Infect Immun. 2021;89(9), e0018721. 10.1128/IAI.00187-21. 33941577 PMC8370678

[18] Starks AM, DiRita VJ, Schoeb TR, Tamplin ML, Parveen S, Doyle TJ, Bomeisl PE, Escudero GM, Gulig PA. Pathogenesis of infection by clinical and environmental strains of Vibrio vulnificus in iron-dextran-treated mice. Infect Immun. 2000;68(10):5785–5793. doi: 10.1128/IAI.68.10.5785-5793.2000. 10992486 PMC101538

[19] Bose D, Saha P, Roy S, Trivedi A, More M, Klimas N, Tuteja A, Chatterjee S. A double-humanized mouse model for studying host gut microbiome-immune interactions in Gulf war illness. Int J Mol Sci. 2024;25(11):6093. doi: 10.3390/ijms25116093. 38892281 PMC11172868

[20] Tirelle P, Breton J, Riou G, Déchelotte P, Coëffier M, Ribet D. Comparison of different modes of antibiotic delivery on gut microbiota depletion efficiency and body composition in mouse. BMC Microbiol. 2020;20(1):340. doi: 10.1186/s12866-020-02018-9. 33176677 PMC7657353

[21] Scheperjans F, Levo R, Bosch B, Lääperi M, Pereira PAB, Smolander O, Aho VTE, Vetkas N, Toivio L, Kainulainen V, et al. Fecal microbiota transplantation for treatment of parkinson disease: a randomized clinical trial. JAMA Neurol. 2024;81(9):925–938. doi: 10.1001/jamaneurol.2024.2305. 39073834 PMC11287445

[22] Giles ED, Cook KL, Jenschke RM, Corleto KA, Landrock D, Mahmood TN, Sanchez KE, Levin A, Hursting SD, Kimler BF, et al. Metabolic and transcriptional effects of bazedoxifene/conjugated estrogens in a model of obesity-associated breast cancer risk. JCI Insight. 2025;10(8). doi: 10.1172/jci.insight.182694. PMC12016928 40048260

[23] Saha P, Bose D, Stebliankin V, Cickovski T, Seth RK, Porter DE, Brooks BW, Mathee K, Narasimhan G, Colwell R, et al. Prior exposure to microcystin alters host gut resistome and is associated with dysregulated immune homeostasis in translatable mouse models. Sci Rep. 2022;12(1):11516. doi: 10.1038/s41598-022-15708-3. 35799048 PMC9262933

[24] Ponnusamy D, Kozlova EV, Sha J, Erova TE, Azar SR, Fitts EC, Kirtley ML, Tiner BL, Andersson JA, Grim CJ, et al. Cross-talk among flesh-eating Aeromonas hydrophila strains in mixed infection leading to necrotizing fasciitis. Proc Natl Acad Sci U S A. 2016;113(3):722–727. doi: 10.1073/pnas.1523817113. 26733683 PMC4725515

[25] Saha P, Bose D, Roy S, More M, Trivedi A, Brooks BW, Xiao S, Syn W, Diehl AM, Chatterjee S. Peroxynitrite is key to Cylindrospermopsin-mediated MASLD to MASH progression via triggering TXNIP binding to NLRP3 and subsequent inflammasome activation. Toxicol Appl Pharmacol. 2025;504:117527. doi: 10.1016/j.taap.2025.117527. 40846139

[26] Saha P, Upright M, Bose D, Roy S, Trivedi A, More M, Scott GI, Brooks BW, Chatterjee S. Subchronic oral cylindrospermopsin exposure alters the host Gut microbiome and is associated with progressive hepatic inflammation, stellate cell activation, and mild fibrosis in a preclinical study. Toxins (Basel). 2022;14(12):835. doi: 10.3390/toxins14120835. 36548732 PMC9785749

[27] Kleiner DE, Brunt EM, Van Natta M, Behling C, Contos MJ, Cummings OW, Ferrell LD, Liu Y, Torbenson MS, Unalp‐Arida A, et al. Design and validation of a histological scoring system for nonalcoholic fatty liver disease. Hepatology. 2005;41(6):1313–1321. doi: 10.1002/hep.20701. 15915461

[28] Daemen S, Chan MM, Schilling JD. Comprehensive analysis of liver macrophage composition by flow cytometry and immunofluorescence in murine NASH. STAR Protoc. 2021;2(2):100511. doi: 10.1016/j.xpro.2021.100511. 33997821 PMC8102804

[29] Roy S, More M, Trivedi A, Saha P, Bose D, Das S, Mahmud ZH, Hanifi SMA, Chatterjee S. Aging and climate change-induced heat stress synergistically increase susceptibility to Vibrio vulnificus infection via an altered gut microbiome-immune axis. Sci Total Environ. 2025;989:179881. doi: 10.1016/j.scitotenv.2025.179881. 40516193

[30] Grondin JA, Kwon YH, Far PM, Haq S, Khan WI, et al. Mucins in Intestinal Mucosal Defense and Inflammation: Learning From Clinical and Experimental Studies. Front Immunol. 2020;11:2054–2054. doi: 10.3389/fimmu.2020.02054. 33013869 PMC7500085

[31] Peng L, Li Z, Green RS, Holzmanr IR, Lin J. Butyrate enhances the intestinal barrier by facilitating tight junction assembly via activation of AMP-activated protein kinase in Caco-2 cell monolayers. J Nutr. 2009;139(9):1619–1625. doi: 10.3945/jn.109.104638. 19625695 PMC2728689

[32] Parada Venegas D, De la Fuente MK, Landskron G, González MJ, Quera R, Dijkstra G, Harmsen HJM, Faber KN, Hermoso MA. Corrigendum: Short Chain Fatty Acids (SCFAs)-Mediated Gut epithelial and immune regulation and its relevance for inflammatory bowel diseases. Front Immunol. 2019;10:1486. doi: 10.3389/fimmu.2019.01486. 31316522 PMC6611342

[33] Johansson ME, Larsson JM, Hansson GC. The two mucus layers of colon are organized by the MUC2 mucin, whereas the outer layer is a legislator of host-microbial interactions. Proc Natl Acad Sci U S A. 2011;108Suppl 1(Suppl 1):4659–4665. doi: 10.1073/pnas.1006451107. PMC3063600 20615996

[34] Johansson ME, Gustafsson JK, Holmén-Larsson J, Jabbar KS, Xia L, Xu H, Ghishan FK, Carvalho FA, Gewirtz AT, Sjövall H, et al. Bacteria penetrate the normally impenetrable inner colon mucus layer in both murine colitis models and patients with ulcerative colitis. Gut. 2014;63(2):281–291. doi: 10.1136/gutjnl-2012-303207. 23426893 PMC3740207

[35] Birchenough GM, Nyström EEL, Johansson MEV, Hansson GC. A sentinel goblet cell guards the colonic crypt by triggering Nlrp6-dependent Muc2 secretion. Science. 2016;352(6293):1535–1542. doi: 10.1126/science.aaf7419. 27339979 PMC5148821

[36] Tacke F, Zimmermann HW. Macrophage heterogeneity in liver injury and fibrosis. J Hepatol. 2014;60(5):1090–1096. doi: 10.1016/j.jhep.2013.12.025. 24412603

[37] Kamari Y, Shaish A, Vax E, Shemesh S, Kandel-Kfir M, Arbel Y, Olteanu S, Barshack I, Dotan S, Voronov E, et al. Lack of interleukin-1α or interleukin-1β inhibits transformation of steatosis to steatohepatitis and liver fibrosis in hypercholesterolemic mice. J Hepatol. 2011;55(5):1086–1094. doi: 10.1016/j.jhep.2011.01.048. 21354232 PMC3210940

[38] Grabherr F, Grander C, Effenberger M, Adolph TE, Tilg H. Gut dysfunction and non-alcoholic fatty liver disease. Front Endocrinol (Lausanne). 2019;10:611. doi: 10.3389/fendo.2019.00611. 31555219 PMC6742694

[39] Senates E, Yilmaz Y, Colak Y, Ozturk O, Altunoz ME, Kurt R, Ozkara S, Aksaray S, Tuncer I, Ovunc AOK. Serum levels of hepcidin in patients with biopsy-proven nonalcoholic fatty liver disease. Metab Syndr Relat Disord. 2011;9(4):287–290. doi: 10.1089/met.2010.0121. 21417913

[40] Makri E, Orfanidou M, Goulas A, Terpos E, Polyzos SA. Circulating ferritin in patients with nonalcoholic fatty liver disease: a systematic review and meta-analysis. J Clin Exp Hepatol. 2024;14(3):101353. doi: 10.1016/j.jceh.2024.101353. 38435724 PMC10905002

[41] Ganz T. Hepcidin and iron regulation, 10 years later. Blood. 2011;117(17):4425–4433. doi: 10.1182/blood-2011-01-258467. 21346250 PMC3099567

[42] Chasteen ND, Harrison PM. Mineralization in ferritin: an efficient means of iron storage. J Struct Biol. 1999;126(3):182–194. doi: 10.1006/jsbi.1999.4118. 10441528

[43] Oppen K, Ueland T, Siljan WW, Skadberg Ø, Brede C, Lauritzen T, Aukrust P, Steinsvik T, Husebye E, Michelsen AE, et al. Hepcidin and ferritin predict microbial etiology in community-acquired pneumonia. Open Forum Infect Dis. 2021;8(4):ofab082. doi: 10.1093/ofid/ofab082. 33880390 PMC8043258

[44] McPherson S, Henderson E, Burt AD, Day CP, Anstee QM. Serum immunoglobulin levels predict fibrosis in patients with non-alcoholic fatty liver disease. J Hepatol. 2014;60(5):1055–1062. doi: 10.1016/j.jhep.2014.01.010. 24445215

[45] Kolodziejczyk AA, Zheng D, Shibolet O, Elinav E. The role of the microbiome in NAFLD and NASH. EMBO Mol Med. 2019;11(2). doi: 10.15252/emmm.201809302. PMC6365925 30591521

[46] Aron-Wisnewsky J, Vigliotti C, Witjes J, Le P, Holleboom AG, Verheij J, Nieuwdorp M, Clément K. Gut microbiota and human NAFLD: disentangling microbial signatures from metabolic disorders. Nat Rev Gastroenterol Hepatol. 2020;17(5):279–297. doi: 10.1038/s41575-020-0269-9. 32152478

[47] Saha P, Hartmann P. Impact of Gut microbiome on Gut permeability in liver and gut diseases. Microorganisms. 2025;13(6):1188. doi: 10.3390/microorganisms13061188. 40572077 PMC12195470

[48] Bazanella M, Maier TV, Clavel T, Lagkouvardos I, Lucio M, Maldonado-Gòmez MX, Autran C, Walter J, Bode L, Schmitt-Kopplin P, et al. Randomized controlled trial on the impact of early-life intervention with bifidobacteria on the healthy infant fecal microbiota and metabolome. Am J Clin Nutr. 2017;106(5):1274–1286. doi: 10.3945/ajcn.117.157529. 28877893

[49] Wang H, He S, Xin J, Zhang T, Sun N, Li L, Ni X, Zeng D, Ma H, Bai Y. Psychoactive Effects of. Front Pharmacol. 2021;12:662148. doi: 10.3389/fphar.2021.662148. 34122081 PMC8189558

[50] Derrien M, Belzer C, de Vos WM. Akkermansia muciniphila and its role in regulating host functions. Microb Pathog. 2017;106:171–181. doi: 10.1016/j.micpath.2016.02.005. 26875998

[51] Desai MS, Seekatz AM, Koropatkin NM, Kamada N, Hickey CA, Wolter M, Pudlo NA, Kitamoto S, Terrapon N, Muller A, et al. A dietary fiber-deprived gut microbiota degrades the colonic mucus barrier and enhances pathogen susceptibility. Cell. 2016;167(5):1339–1353.e21. doi: 10.1016/j.cell.2016.10.043. 27863247 PMC5131798

[52] Qu S, Zheng Y, Huang Y, Feng Y, Xu K, Zhang W, Wang Y, Nie K, Qin M. Excessive consumption of mucin by over-colonized. Front Microbiol. 2023;14:1111911. doi: 10.3389/fmicb.2023.1111911. 36937258 PMC10018180

[53] Xu J, Bjursell MK, Himrod J, Deng S, Carmichael LK, Chiang HC, Hooper LV, Gordon JI. A genomic view of the human-Bacteroides thetaiotaomicron symbiosis. Science. 2003;299(5615):2074–2076. doi: 10.1126/science.1080029. 12663928

[54] Wexler HM. Bacteroides: the good, the bad, and the nitty-gritty. Clin Microbiol Rev. 2007;20(4):593–621. doi: 10.1128/CMR.00008-07. 17934076 PMC2176045

[55] Liang W, Menke AL, Driessen A, Koek GH, Lindeman JH, Stoop R, Havekes LM, Kleemann R, van den Hoek AM, Sookoian SC. Establishment of a general NAFLD scoring system for rodent models and comparison to human liver pathology. PLoS One. 2014;9(12):e115922. doi: 10.1371/journal.pone.0115922. 25535951 PMC4275274

