# Supplementary material for: Underlying MASLD-induced gut microbiome dysbiosis and intestinal inflammation are key to poor outcomes in vibriosis infections in a preclinical model
Source: Gut Microbes. 2026 Apr 13;18(1):2652474. doi: 10.1080/19490976.2026.2652474 (PMC13078222; doi:10.1080/19490976.2026.2652474)
Supplement: MASLD VV Supplementary File Revised.docx [file KGMI_A_2652474_SM9783.docx]

**Underlying MASLD-induced gut microbiome dysbiosis and intestinal inflammation are key to poor outcomes in vibriosis infections in a preclinical model.**

Punnag Saha, Subhajit Roy, Madhura More, Dipro Bose, Ayushi Trivedi, Bryan W. Brooks, Wing-Kin Syn, Anna Mae Diehl, and Saurabh Chatterjee.

Table of Contents

[Supplementary Table 1. 2](#_Toc220529602)

[Supplementary Figure S1 3](#_Toc220529603)

[Supplementary Figure S2. 4](#_Toc220529604)

[Supplementary Figure S3. 6](#_Toc220529605)

[Supplementary Figure S4. 8](#_Toc220529606)

**Supplementary Table 1. List of Primary and Secondary Antibodies.**

| **Antibody** | **Manufacturer** | **Catalog No.** |
| --- | --- | --- |
| Alpha Smooth Muscle Actin (α-SMA) | Abcam | AB5694 |
| Anti-Mouse IgG, HRP-linked Antibody | Cell Signaling Technology | 7076S |
| Anti-Mouse Secondary IgG Antibody, Biotinylated | Vector Laboratories | BA-9200 |
| Anti-Rabbit IgG (H+L) Secondary Antibody, Alexa Fluor™ 488 | Invitrogen | A11008 |
| Anti-Rabbit IgG Secondary Antibody, Alexa Fluor™ 633 | Invitrogen | A21071 |
| Anti-Rabbit IgG, HRP-linked Antibody | Cell Signaling Technology | 7074S |
| Anti-Rabbit Secondary IgG Antibody, Biotinylated | Vector Laboratories | BA-1000 |
| Claudin-2 | Abcam | AB53032 |
| Fibronectin | Santacruz Biotechnology | sc-52331 |
| Horizon™ BUV395 Rat Anti-Mouse CD45 | BD Biosciences | 564279 |
| Horizon™ BV711 Rat Anti-Mouse F4/80 | BD Biosciences | 565612 |
| Horizon™ V450 Rat anti-CD11b | BD Biosciences | 560455 |
| Interleukin-1 beta (IL-1β) | Santacruz Biotechnology | sc-52012 |
| Mucin 2 (MUC2) | Proteintech | 27675-I-AP |
| Occludin | Abcam | AB216327 |
| PE anti-mouse CD86 | BioLegend | 105008 |
| TGF beta 1 (TGF-β1) | Santacruz Biotechnology | sc-130348 |
| Zonula Occludens-1 (ZO-1) | Proteintech | 21773-1-AP |

# **Supplementary Figure S1**


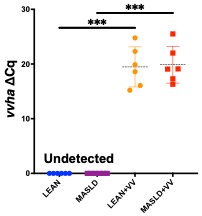


Quantification of the *vvha* gene by qRT-PCR in the fecal samples obtained from LEAN [n=6; Chow diet-fed mice for 20 weeks], MASLD [n=6; CD-HFD fed mice for 20 weeks], LEAN+VV [n=6; mice fed with Chow diet for 20 weeks and received oral VV inoculation for 24 hours], and MASLD+VV [n=6; mice fed with CD-HFD for 20 weeks and received oral VV inoculation for 24 hours] mouse groups post-infection. Data were represented as mean±SEM, and statistical significance was tested using one-way ANOVA between all the groups, followed by Bonferroni Post hoc corrections (**p*<0.05, ***p*<0.01, ****p*<0.001).

# **Supplementary Figure S2.**


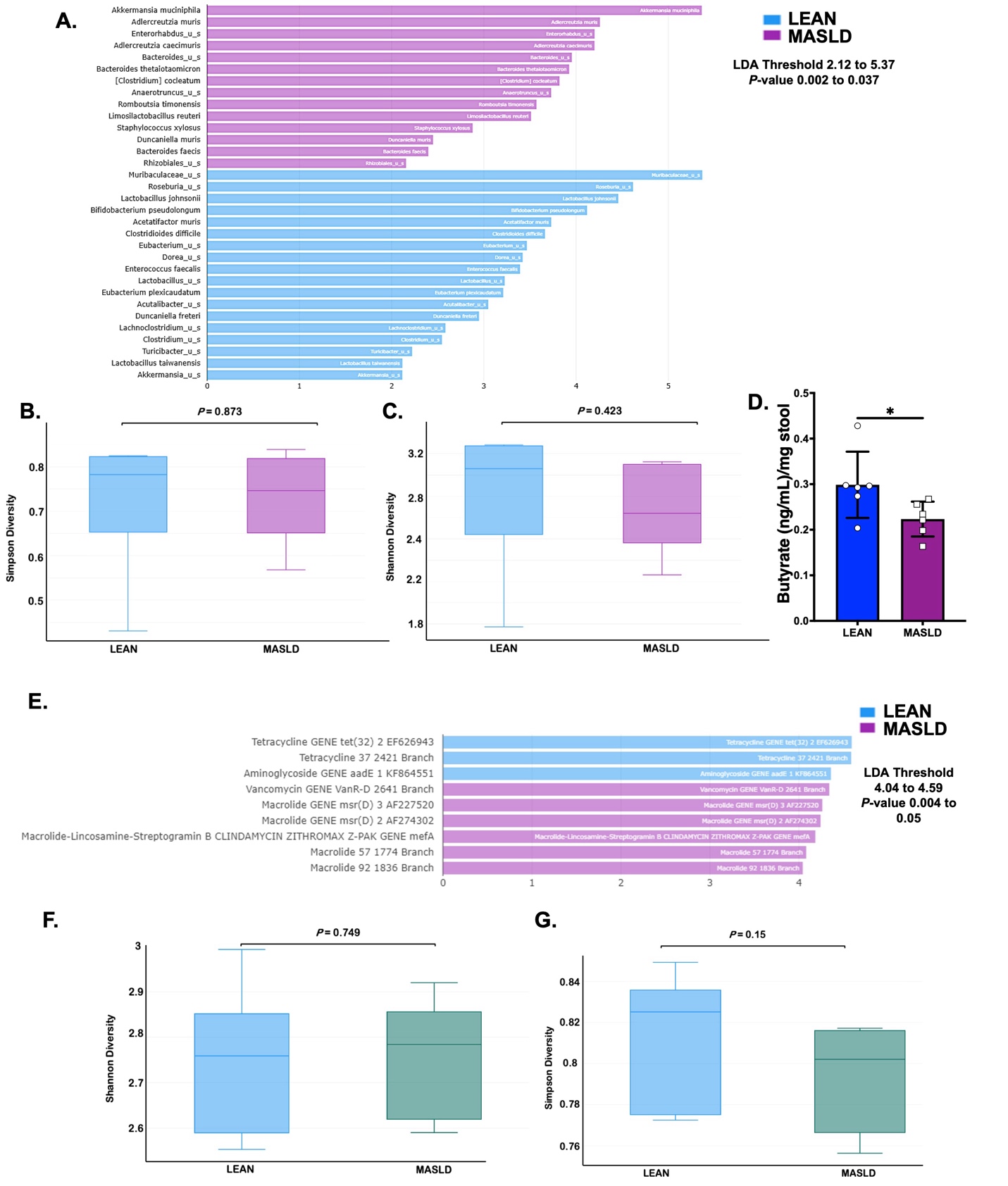


**(A.)** LEfSe (Linear Discriminant Analysis Effect Size) bar chart representing the gut bacteriome in the LEAN [n=6; Chow diet-fed mice for 20 weeks] and MASLD [n=6; CD-HFD fed mice for 20 weeks] mouse groups. Box plot depicting **(B.)** Simpson diversity (*p*=0.873), and **(C.)** Shannon diversity (*p*=0.423) of the gut bacteriome in the LEAN and MASLD mouse groups. **(D.)** Fecal butyrate levels (ng/mL per mg of stool) in the LEAN and MASLD mouse groups. **(E.)** LEfSe bar chart representing the LEAN and MASLD mouse groups. Box plot depicting **(F.)** Simpson diversity (*p*=0.15), and **(G.)** Shannon diversity (*p*=0.749). For diversity indices, statistical significance was tested using the Wilcoxon Rank Sum Test. For fecal butyrate levels, data were represented as mean±SEM, and statistical significance was tested using unpaired t-test between the two groups, followed by Bonferroni-Dunn Post hoc corrections (**p*<0.05).

# **Supplementary Figure S3.**


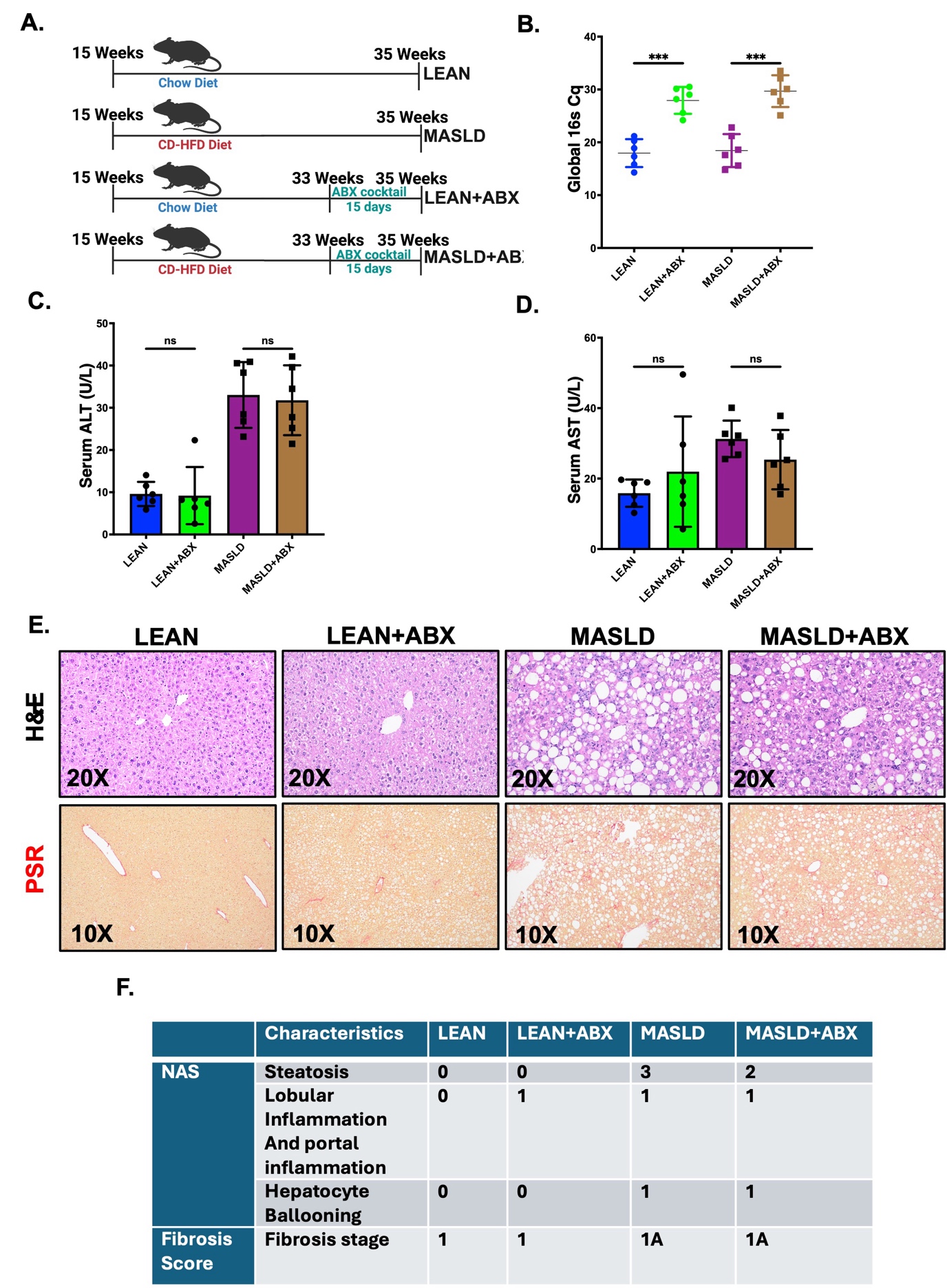


**Depletion of gut microbiota by antibiotic cocktail (ABX) treatment for 15 days did not cause significant liver injury.** **(A.)** Schematic representation of the experimental mouse groups: LEAN [n=6; mice fed with Chow diet for 20 weeks], LEAN+ABX [n=6; Chow diet-fed mice, treated with ABX cocktail for 15 days], MASLD [n=6; mice fed with CD-HFD for 20 weeks], and MASLD+ABX [n=6; CD-HFD fed mice, treated with ABX cocktail for 15 days]. **(B.)** qRT-PCR of the Global 16S gene, serum levels of **(C.)** ALT (U/L), **(D.)** AST (U/L) in LEAN, LEAN+ABX, MASLD, and MASLD+ABX mouse groups. Formalin-fixed, paraffin-embedded 5 μm liver slices were used for histopathological analyses. Representative images of **(E.)** Hematoxylin and Eosin (H&E) staining and Picrosirius red (PSR) staining in the liver sections of LEAN, LEAN+ABX, MASLD, and MASLD+ABX mouse groups. H&E images were captured at 20× magnification, whereas PSR images were captured at 10× magnification. **(F.)** NAFLD activity score (NAS) and fibrosis score for LEAN, LEAN+ABX, MASLD, and MASLD+ABX mouse groups. Data were represented as mean±SEM, and statistical significance was tested using one-way ANOVA between all the groups, followed by Bonferroni Post hoc corrections (**p*<0.05, ***p*<0.01, ****p*<0.001).

# **Supplementary Figure S4.**


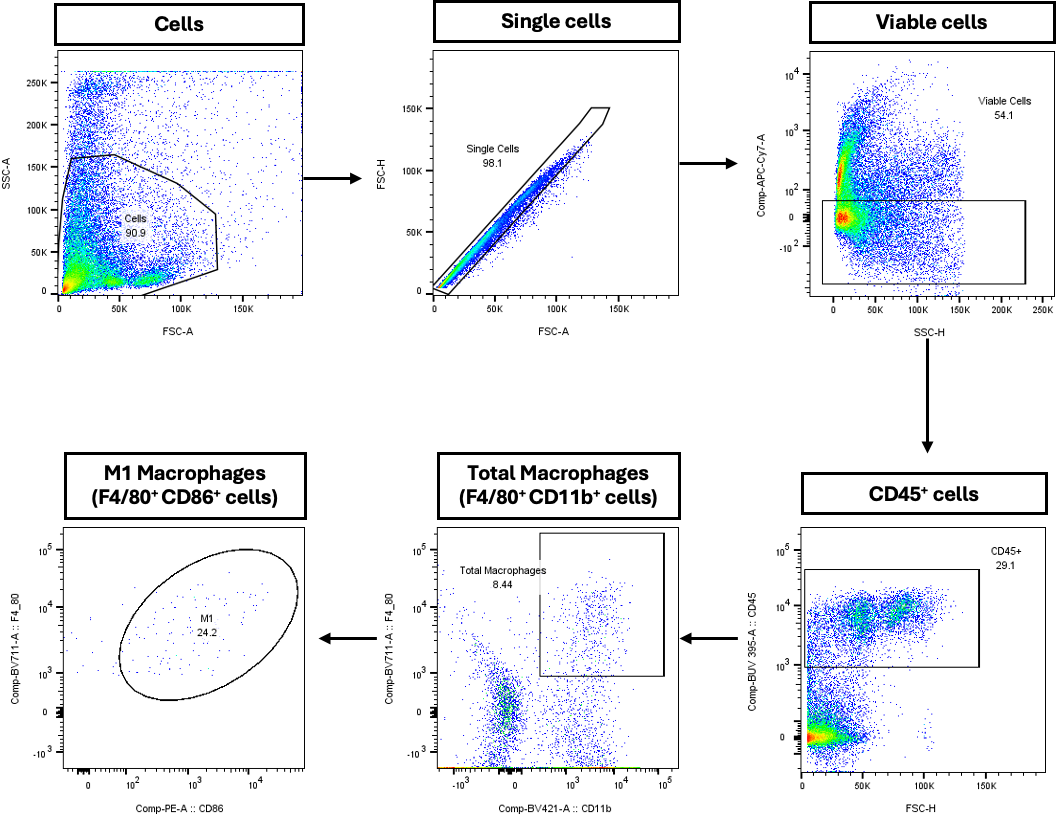


**Flow Cytometry gating strategy to identify hepatic M1 macrophage (CD45^+^F4/80^+^CD11b^+^CD86^+^) population.** Hepatic macrophages were gated based on forward scatter area (FSC-A) vs. side scatter area (SSC-A), and only single cells (98.1%) were selected by gating on forward scatter height (FSC-H) vs. FSC-A. Only viable and single cells were then identified using side scatter height (SSC-H) and APC-Cy7 (live/dead), with a clearly defined population (54.1%). Subsequently, the CD45⁺ cell population was assessed using BUV395 fluorescence. Quadrant gating was applied to distinguish the total macrophage population (F4/80^+^CD11b^+^) from the CD45⁺ cell population, and subsequently, the M1 macrophage population (F4/80^+^CD86^+^) was detected from the total macrophage population. All percentages were calculated as a proportion of the parent population for each population of interest.
